# Supplementary figures and images for: Inferring spikes from calcium imaging in dopamine neurons
Source: PLoS One. 2021 Jun 4;16(6):e0252345. doi: 10.1371/journal.pone.0252345 (PMC8177503; doi:10.1371/journal.pone.0252345)

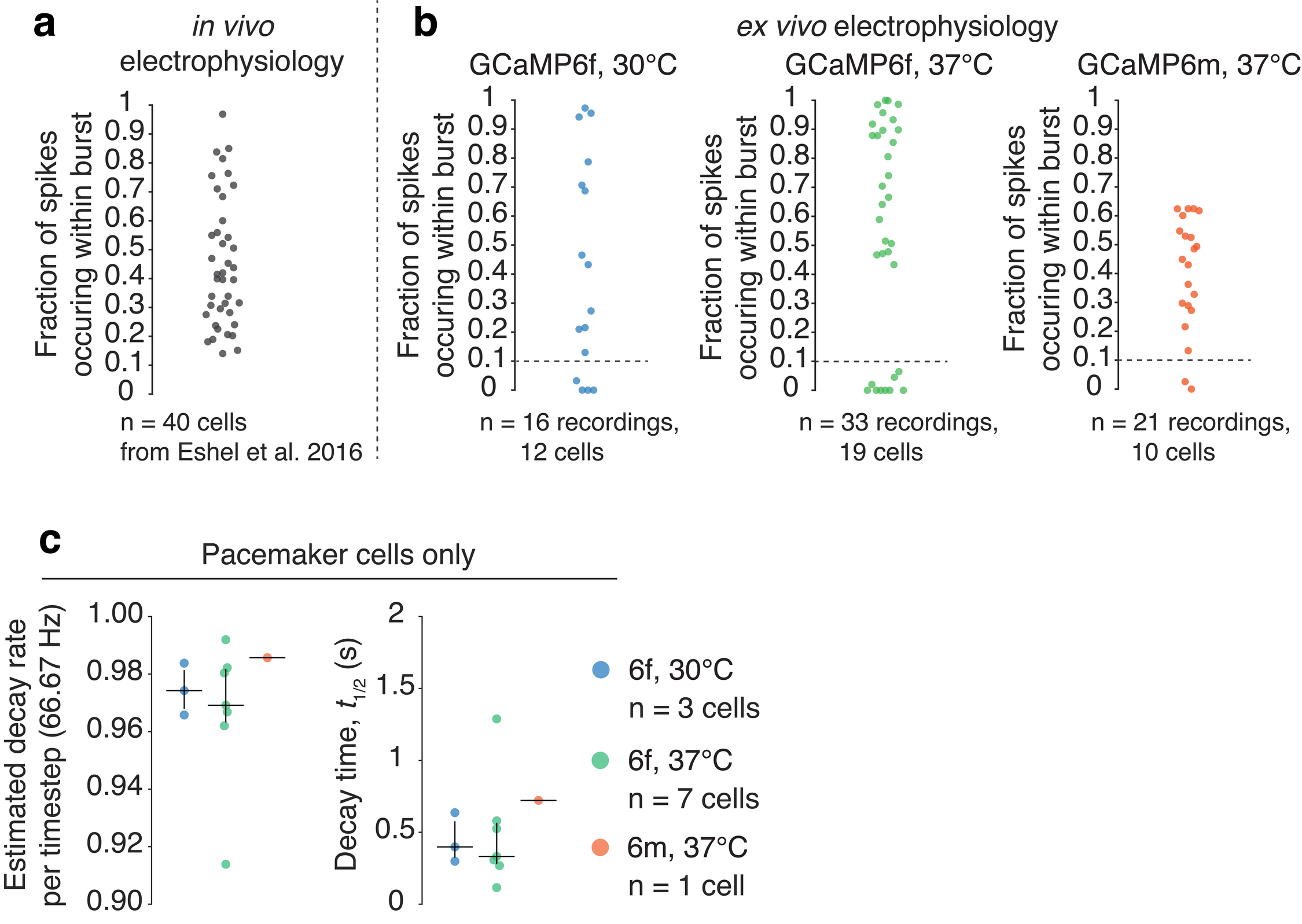

Supplement: S1 Fig — a. Percent of spikes that occurred within a burst in a previously collected in vivo electrophysiology dataset (Eshel et al. [39]). A burst was defined by a series of at least 3 spikes with < = 100 ms between each spike. All in vivo electrophysiology cells had >10% of spikes within a burst. This was chosen as the cut-off for inclusion in in vitro analysis. b. Percent of spikes that occurred within a burst for all recordings in the GCaMP6f at 30°C, GCaMP6f at 37°C, and GCaMP6m at 37°C groups. Cells with <10% of spikes occurring within a burst were excluded from group analysis, since they featured little or no phasic activity. c. Left: Estimated decay rates for pacemaker cells for GCaMP6f at 30°C (n = 3 cells; median decay rate = 0.974, Q1 = 0.968, Q3 = 0.981), GCaMP6f at 37°C (n = 7 cells; median decay rate = 0.969, Q1 = 0.963, Q3 = 0.982), and GCaMP6m at 37°C (n = 1 cell; decay rate = 0.986). Right: Half decay time for pacemaker cells for GCaMP6f at 30°C (median = 0.398 s, Q1 = 0.323 s, Q3 = 0.577 s), GCaMP6f at 37°C (median = 0.332 s, Q1 = 0.278 s, Q3 = 0.566 s) and GCaMP6m at 37°C (0.722 s). (TIF) [file pone.0252345.s001.tif]

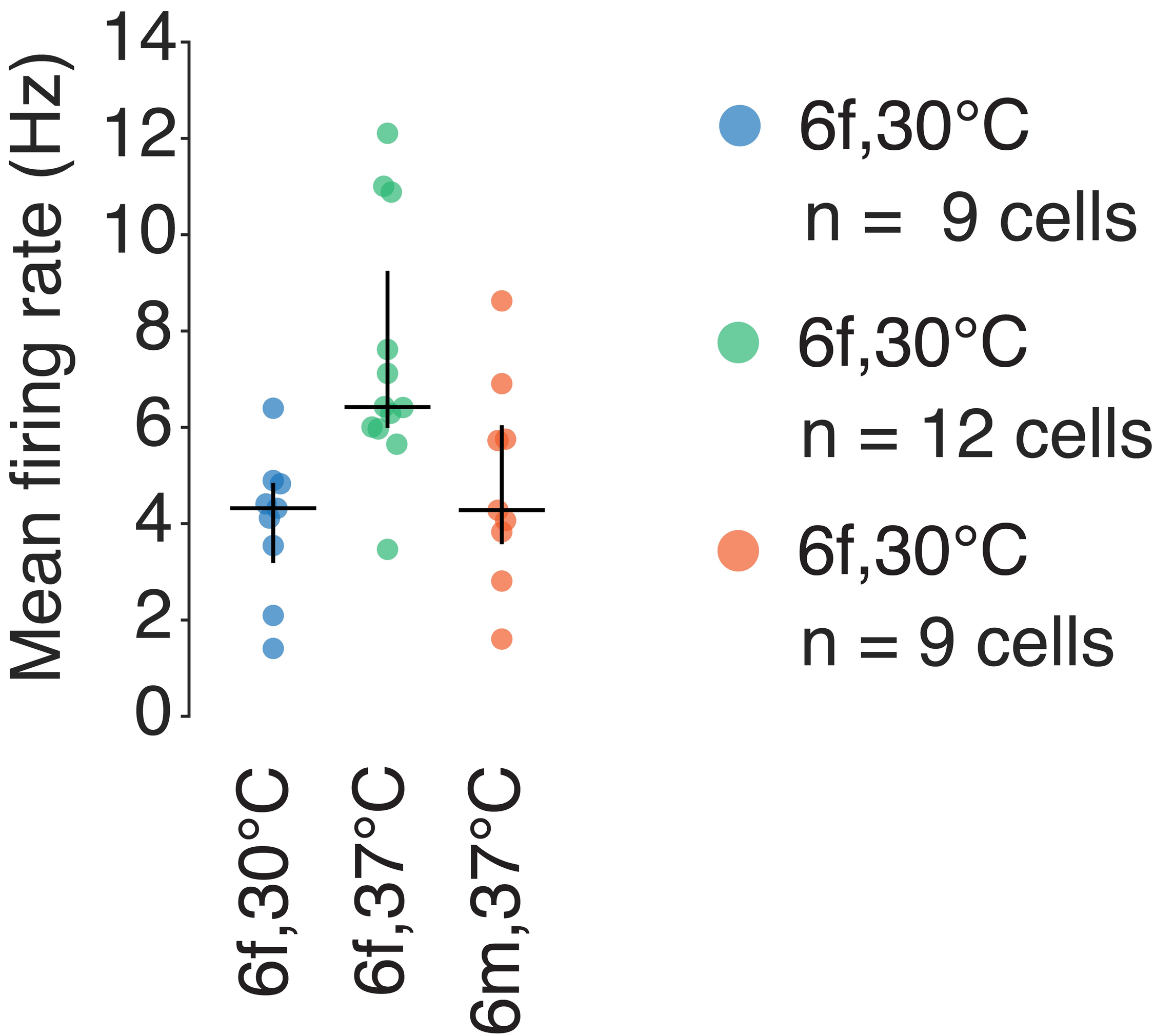

Supplement: S2 Fig — Mean spike frequency during the simultaneous calcium imaging and electrophysiology in vitro experiment (GCaMP6f at 30°C, n = 12 recordings from 9 total cells, cell median firing rate = 4.3 Hz, Q1 = 3.2 Hz, Q3 = 4.8 Hz; GCaMP6f at 37°C, n = 9 recordings from 12 total cells, cell median firing rate = 6.4 Hz, Q1 = 6.0 Hz, Q3 = 9.3 Hz; GCaMP6m at 37°C, n = 19 recordings from 9 total cells, cell median firing rate = 4.2 Hz, Q1 = 3.6 Hz, Q3 = 6.0 Hz). Vertical bars are interquartile range (Q1 and Q3). (TIF) [file pone.0252345.s002.tif]

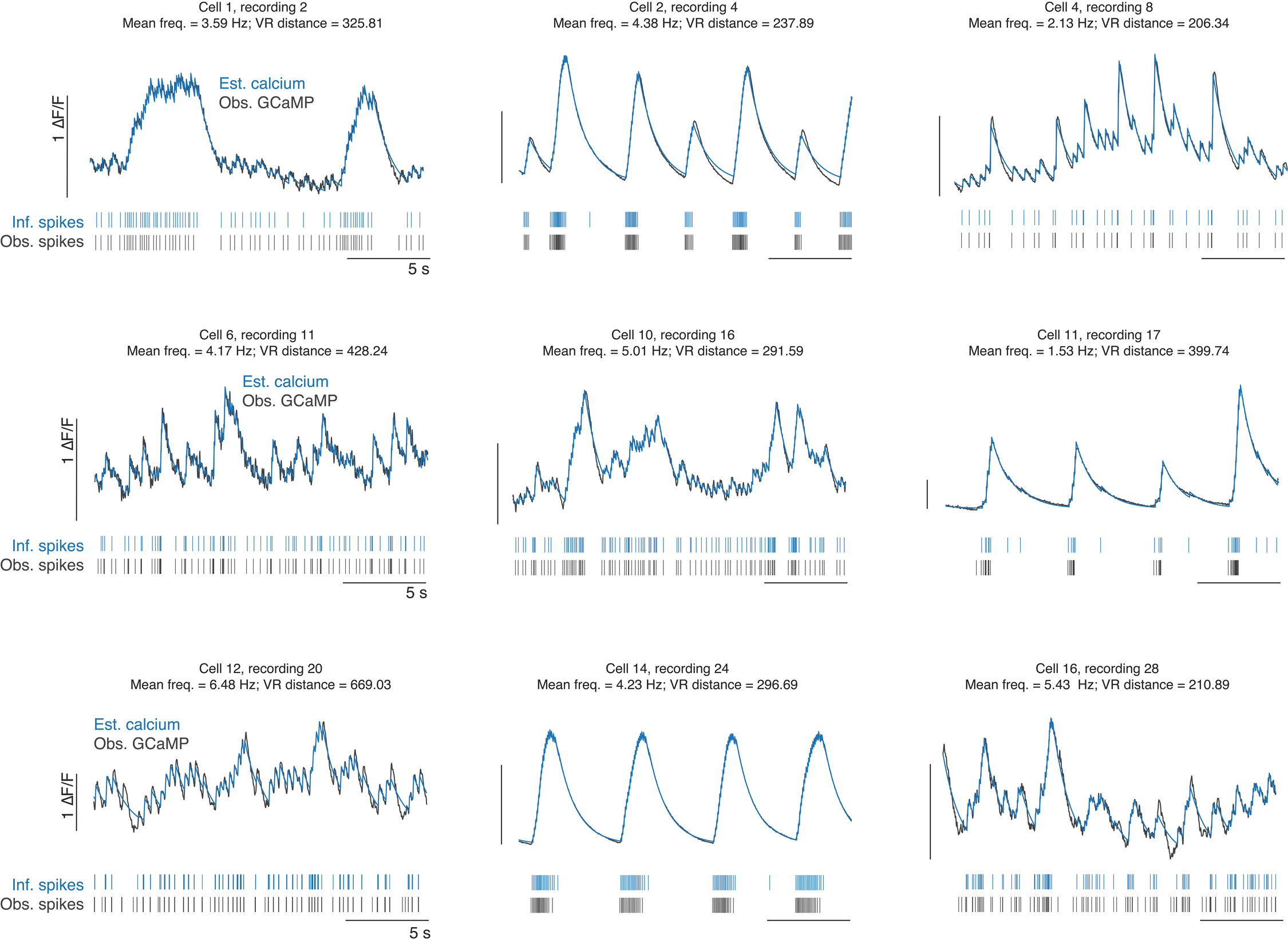

Supplement: S3 Fig — Examples of correspondence between observed GCaMP (black line, top) and estimated calcium (blue line, top), and observed spikes (black dashes, bottom) and inferred spikes (blue dashes, bottom) for each recording in the GCaMP6f 30°C group. Traces are 20 s long. Scale bars are 1 ΔF/F and 5 s. (TIF) [file pone.0252345.s003.tif]

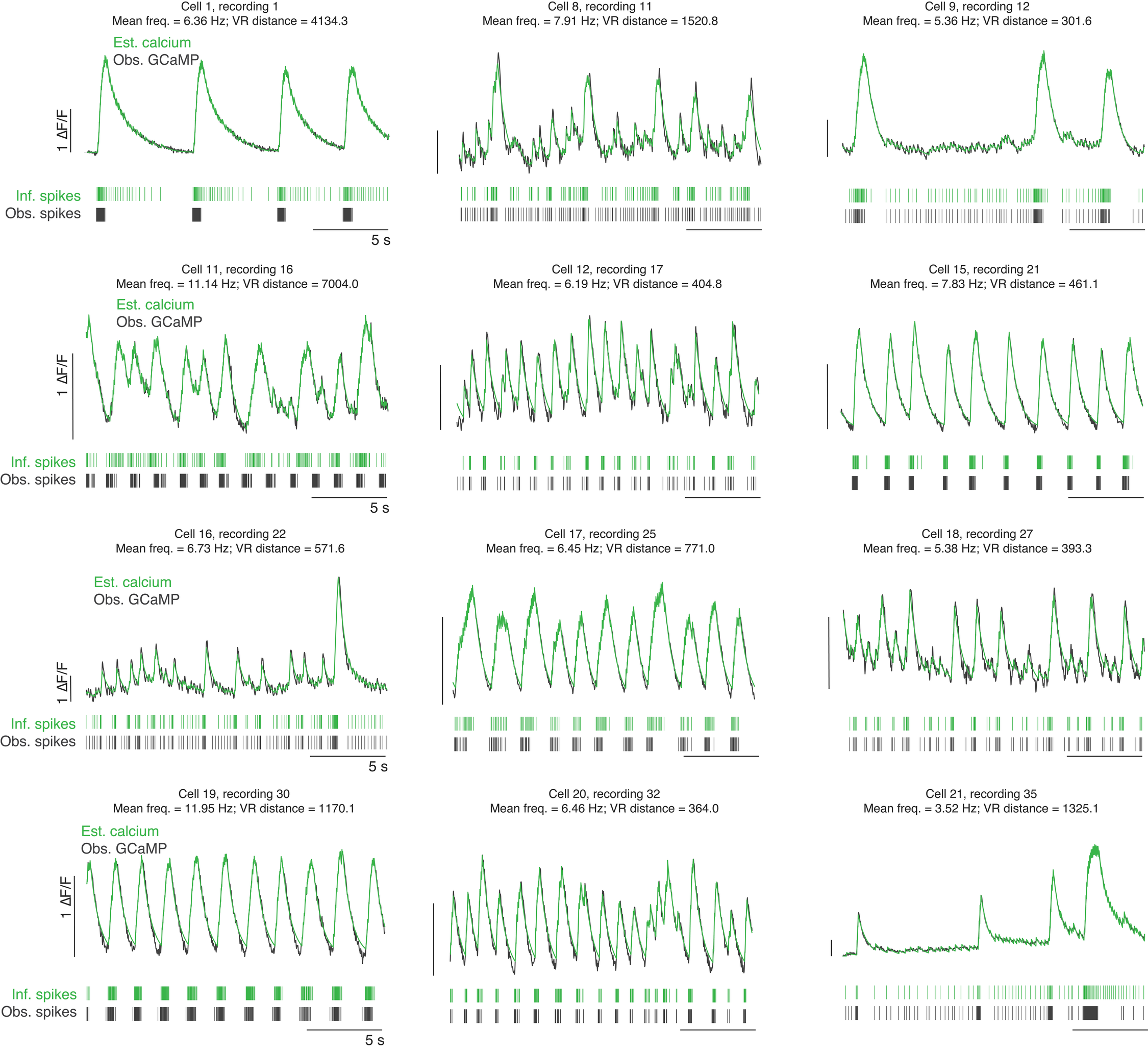

Supplement: S4 Fig — Examples of correspondence between observed GCaMP (black line, top) and estimated calcium (green line, top), and observed spikes (black dashes, bottom) and inferred spikes (green dashes, bottom) for each recording in the GCaMP6f 37°C group. Traces are 20 s long. Scale bars are 1 ΔF/F and 5 s. (TIF) [file pone.0252345.s004.tif]

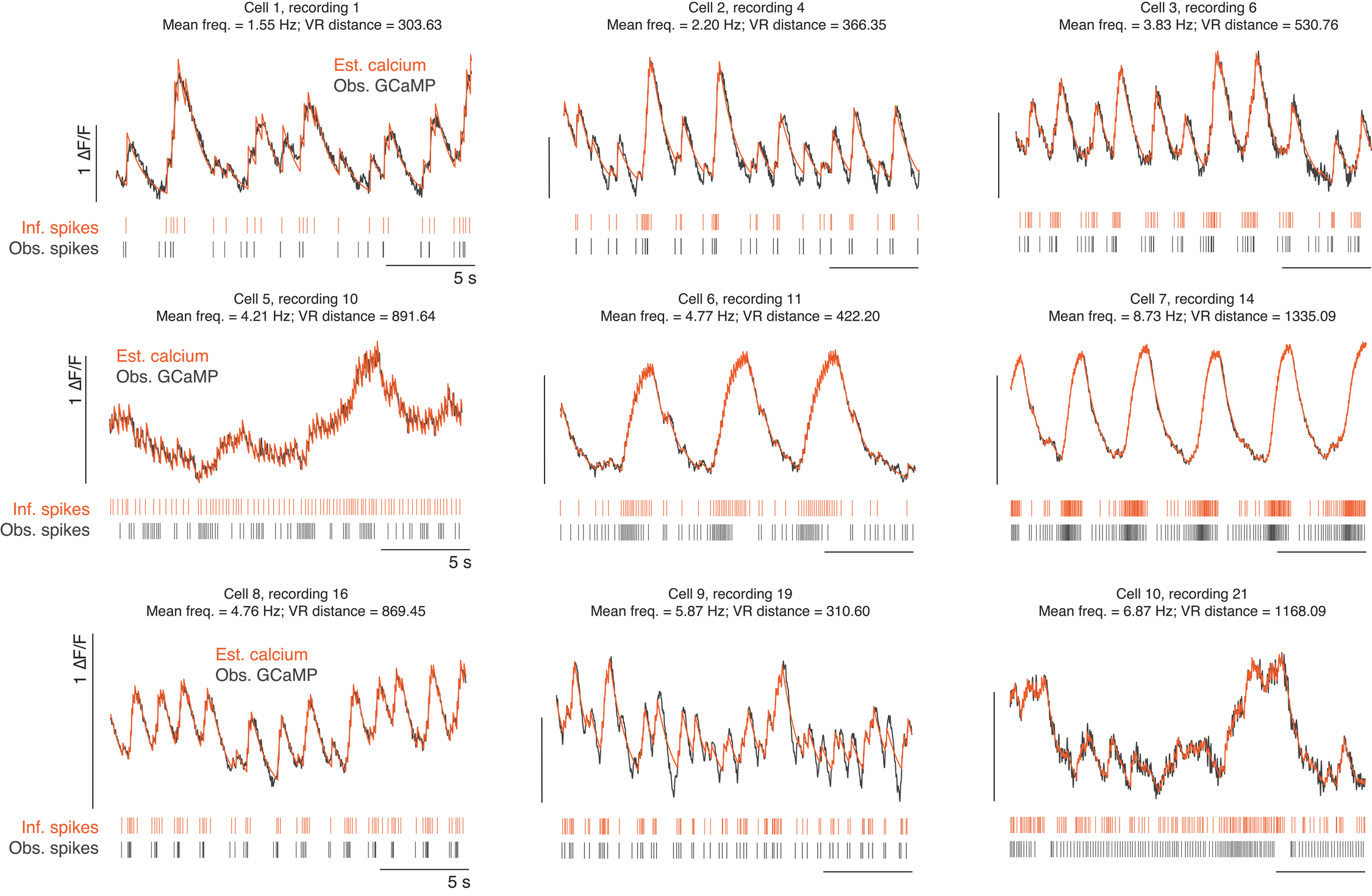

Supplement: S5 Fig — Examples of correspondence between observed GCaMP (black line, top) and estimated calcium (orange line, top), and observed spikes (black dashes, bottom) and inferred spikes (orange dashes, bottom) for each recording in the GCaMP6m 37°C group. Traces are 20 s long. Scale bars are 1 ΔF/F and 5 s. (TIF) [file pone.0252345.s005.tif]

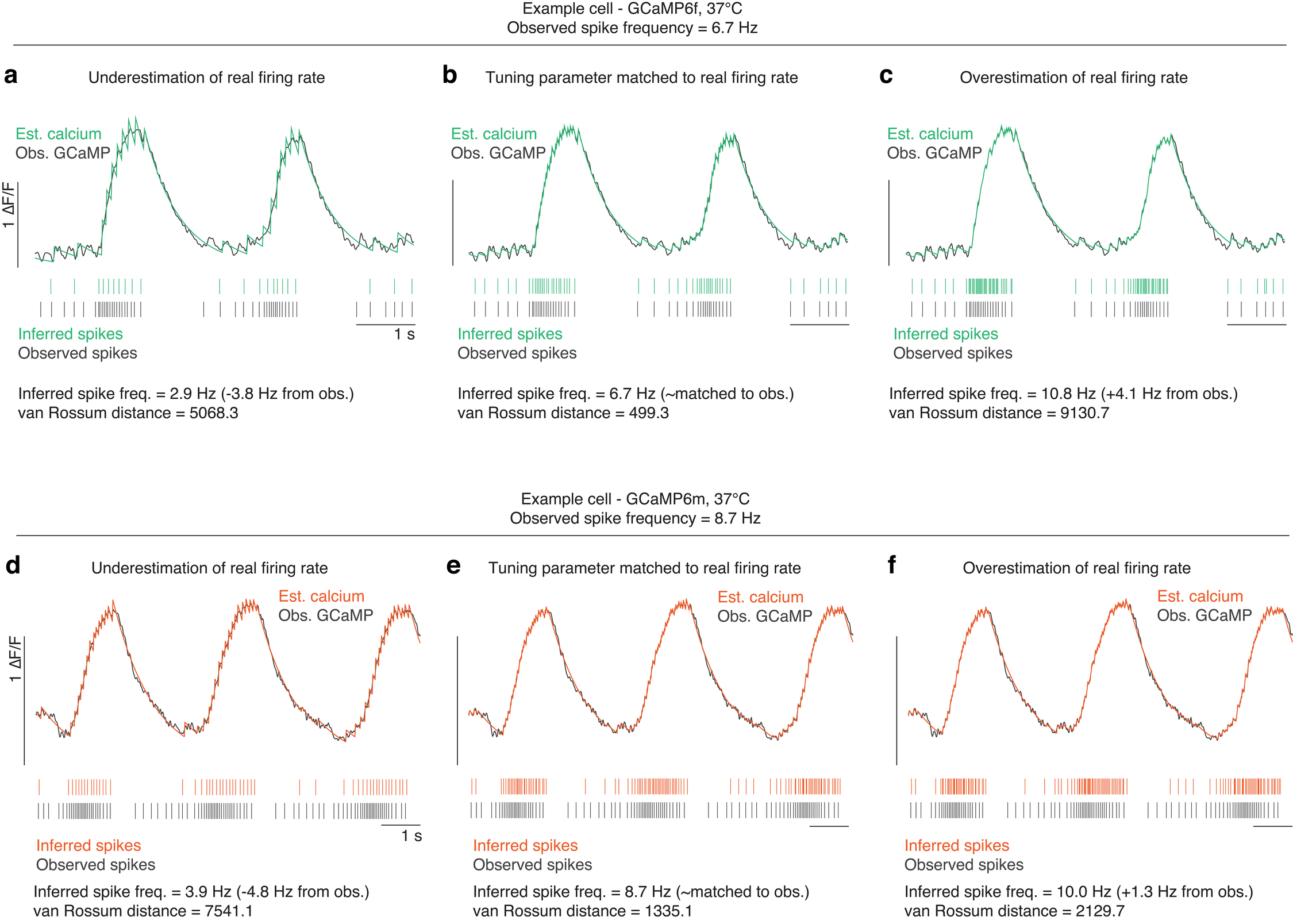

Supplement: S6 Fig — a-c. Examples of spike inference when tuning parameters are selected to underestimate (a), match (b), or overestimate (c) the observed firing rate of a neuron expressing GCaMP6f. d-f. Examples of spike inference when tuning parameters are selected to underestimate (d), match (e), or overestimate (f) the observed firing rate of a neuron expressing GCaMP6m. In both GCaMP6f and GCaMP6m examples, the van Rossum distance is lowest when the tuning parameter is selected to match the firing rate, but under- and overestimates appear to capture both tonic and phasic activity patterns. (TIF) [file pone.0252345.s006.tif]

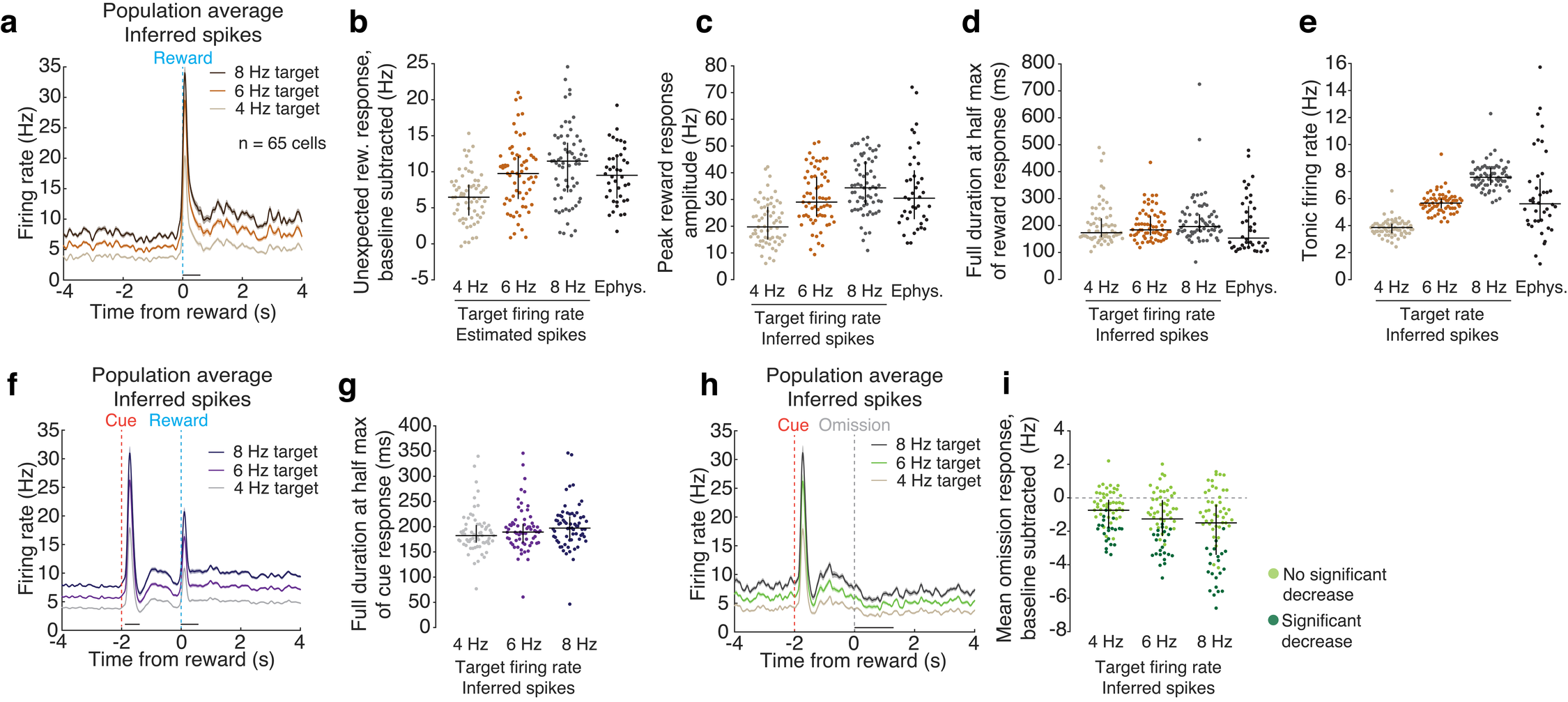

Supplement: S7 Fig — a. Mean population firing rates from inferred spikes around presentation of unexpected reward when λ is selected to target a 4, 6, or 8 Hz average estimated firing rate (n = 65 cells). b-e. Comparison of inferred spikes over a range of λ values and electrophysiology spikes from Eshel et al. [39]. b. Unexpected reward response, where reward response is the mean firing rate over the first 600 ms following reward presentation, baseline subtracted using the mean firing rate over a 1 s period before reward presentation (inferred spikes over baseline median = 6.5 Hz, Q1 = 3.9 Hz, Q3 = 8.2 Hz for λ target 4 Hz; median = 9.8 Hz, Q1 = 6.2 Hz, Q3 = 12.2 Hz, for λ target 6 Hz; median = 11.5 Hz, Q1 = 7.1 Hz, Q3 = 14.0 Hz, for λ target 8 Hz; electrophysiology spikes over baseline median = 9.5 Hz, Q1 = 6.3 Hz, Q3 = 12.0 Hz). c. Peak reward response amplitude in inferred and electrophysiology spikes, where peak is maximum value of PSTH in the first 600 ms period following reward presentation (inferred spikes median = 19.7 Hz, Q1 = 15.0 Hz, Q3 = 27.2 Hz, for λ target 4 Hz; median = 29.0 Hz, Q1 = 23.7 Hz, Q3 = 38.6 Hz, for λ target 6 Hz; median = 34.4 Hz, Q1 = 28.1 Hz, Q3 = 44.3 Hz, for λ target 8 Hz; electrophysiology median = 30.5 Hz, Q1 = 22.6 Hz, Q3 = 40.9 Hz). d. Full duration at half max of reward response peak in inferred and electrophysiology spikes (inferred spikes median full duration at half max = 173.4 ms, Q1 = 156.8 ms, Q3 = 226.8 ms for λ target 4 Hz; median = 183.9 ms, Q1 = 163.6 ms, Q3 = 235.9 ms for λ target 6 Hz; median = 196.2 ms, Q1 = 175.8 ms, Q3 = 242.0 ms for λ target 8 Hz; electrophysiology median full duration at half max = 153.5 ms, Q1 = 120.4 ms, Q3 = 272.5 ms). e. Mean tonic firing rates prior to presentations of unexpected reward, where tonic firing rates are calculated as the mean firing rate over a 1 s period prior to reward presentation (inferred spikes tonic firing rate median = 3.9 Hz, Q1 = 3.4 Hz, Q3 = 4.1 Hz, for λ target 4 Hz; median = 5.7 Hz, Q1 [file pone.0252345.s007.tif]

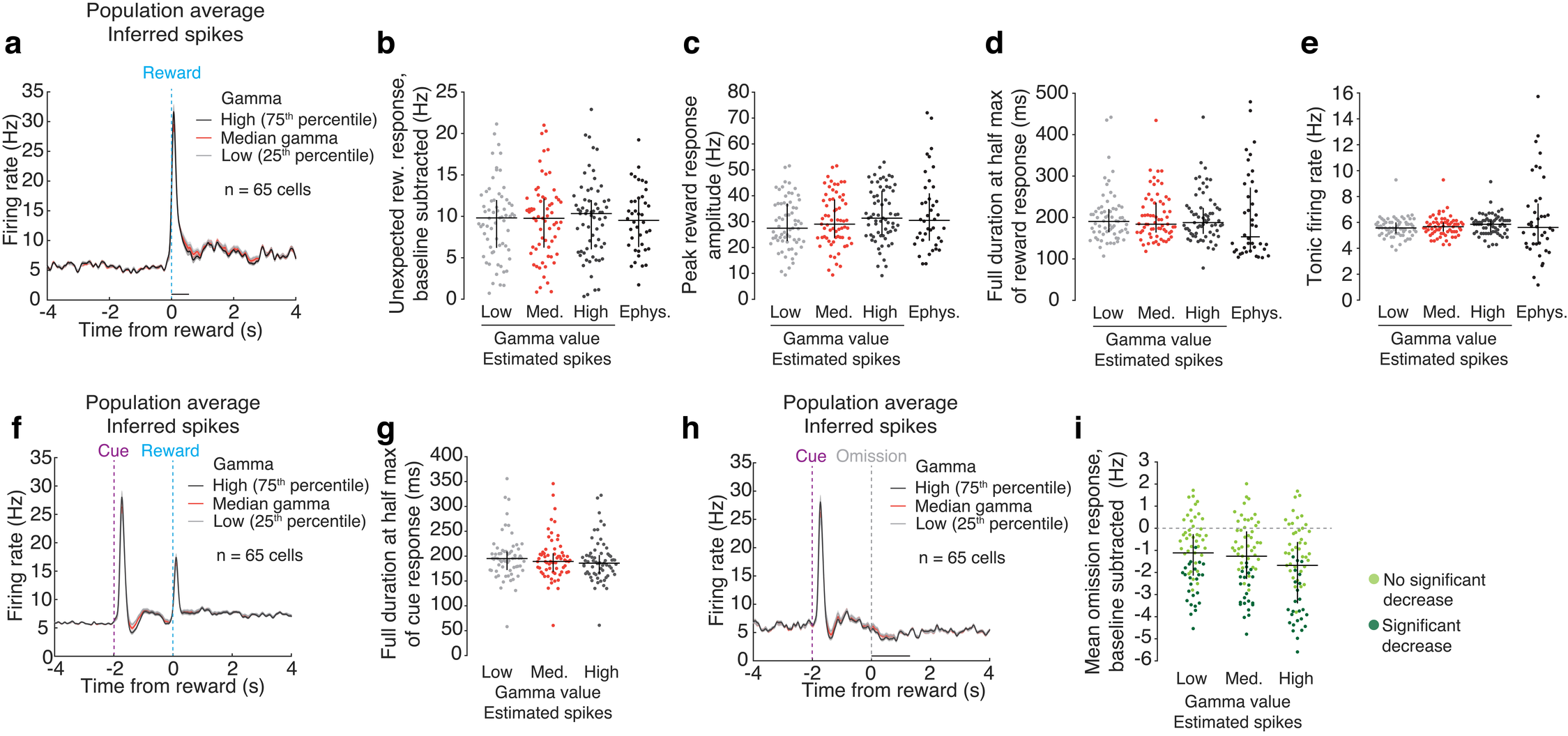

Supplement: S8 Fig — a. Mean population firing rates (n = 65 cells) from inferred spikes around presentation of unexpected reward when λ is selected to target a 6 Hz average estimated firing rate and the decay rate γ is selected as the 25th percentile (low gamma), median, or 75th percentile (high gamma) estimated decay rate from the in vitro experiment. b-e. Comparison of inferred spikes over a range of γ values and electrophysiology spikes from Eshel et al. [39]. b. Unexpected reward response, where reward response is the mean firing rate over the first 600 ms following reward presentation, baseline subtracted using the mean firing rate over a 1 s period before reward presentation (inferred spikes over baseline median = 9.8 Hz, Q1 = 6.2 Hz, Q3 = 12.0 Hz for low gamma; median = 9.8 Hz, Q1 = 6.2 Hz, Q3 = 12.2 Hz, for median gamma; median = 10.3 Hz, Q1 = 6.0 Hz, Q3 = 12.0 Hz, for high gamma; electrophysiology spikes over baseline median = 9.5 Hz, Q1 = 6.3 Hz, Q3 = 12.0 Hz). c. Peak reward response amplitude in inferred and electrophysiology spikes, where peak is maximum value of PSTH in the first 600 ms period following reward presentation (inferred spikes median = 27.5 Hz, Q1 = 22.5 Hz, Q3 = 36.9 Hz for low gamma; median = 29.0 Hz, Q1 = 23.7 Hz, Q3 = 38.6 Hz for median gamma; median = 31.3 Hz, Q1 = 24.8 Hz, Q3 = 41.9 Hz for high gamma; electrophysiology median = 30.5 Hz, Q1 = 22.6 Hz, Q3 = 40.9 Hz). d. Full duration at half max of reward response peak in inferred and electrophysiology spikes (inferred spikes median full duration at half max = 190.6 ms, Q1 = 164.8 ms, Q3 = 220.3 ms for low gamma; median = 183.9 ms, Q1 = 163.6 ms, Q3 = 235.9 ms for median gamma; median = 187.6 ms, Q1 = 160.5 ms, Q3 = 223.3 ms for high gamma; electrophysiology median full duration at half max = 153.5 ms, Q1 = 120.4 ms, Q3 = 272.5 ms). e. Mean tonic firing rates prior to presentations of unexpected reward, where tonic firing rates are calculated as the mean firing rate over a 1 s period prior to reward prese [file pone.0252345.s008.tif]

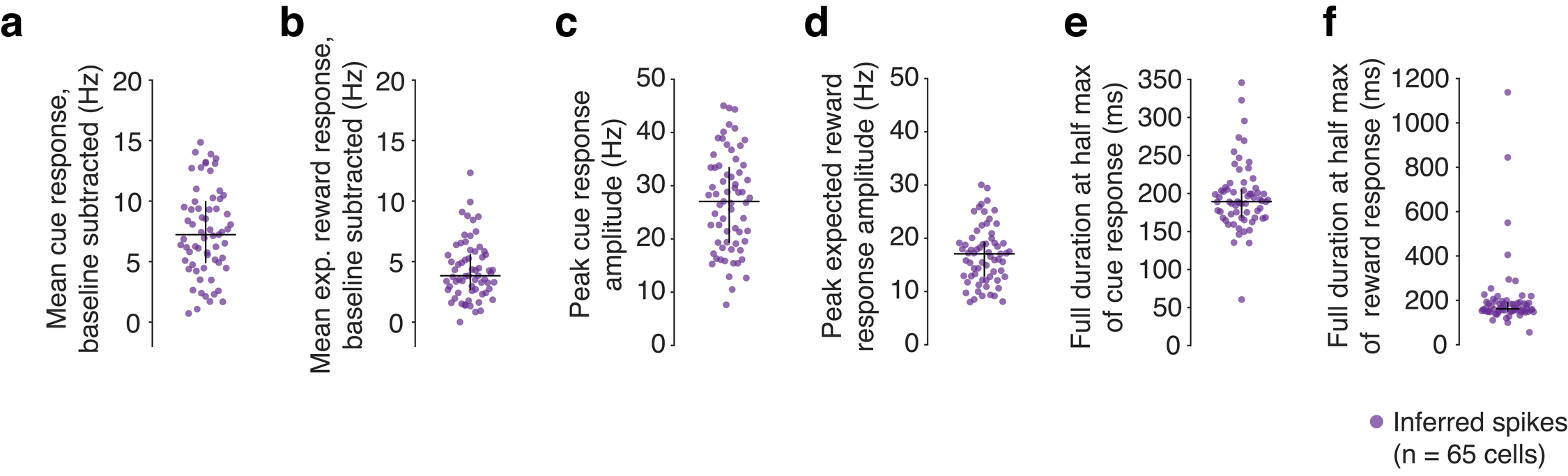

Supplement: S9 Fig — a. Mean cue response, where cue response is the mean firing rate in a 500 ms period beginning 150 ms after cue onset, baseline subtracted using the mean firing rate over a 1 s period before cue (median = 7.2 Hz, Q1 = 4.9 Hz, Q3 = 10.0 Hz). b. Mean expected reward response, where reward response is the mean firing rate over the first 600 ms following reward presentation, baseline subtracted using the mean firing rate over a 1 s period before cue (median = 3.8 Hz, Q1 = 2.7 Hz, Q3 = 5.6 Hz). c. Peak cue response amplitude in inferred spikes, where peak is maximum value of PSTH in the 500 ms cue period (median = 27.0 Hz, Q1 = 19.3 Hz, Q3 = 33.5 Hz). d. Peak expected reward response amplitude in inferred spikes, where peak is maximum value of PSTH in the first 600 ms period following reward presentation (median = 17.1 Hz, Q1 = 12.8 Hz, Q3 = 19.3 Hz). e. Full duration at half max of cue response in inferred spikes (median = 189.3 ms, Q1 = 168.3 ms, Q3 = 205.6 ms). f. Full duration at half max of expected reward response in inferred spikes (median = 162.0 ms, Q1 = 150.0 ms, Q3 = 199.2 ms). Vertical bars are interquartile range (Q1 and Q3). All data from cells expressing GCaMP6f. (TIF) [file pone.0252345.s009.tif]

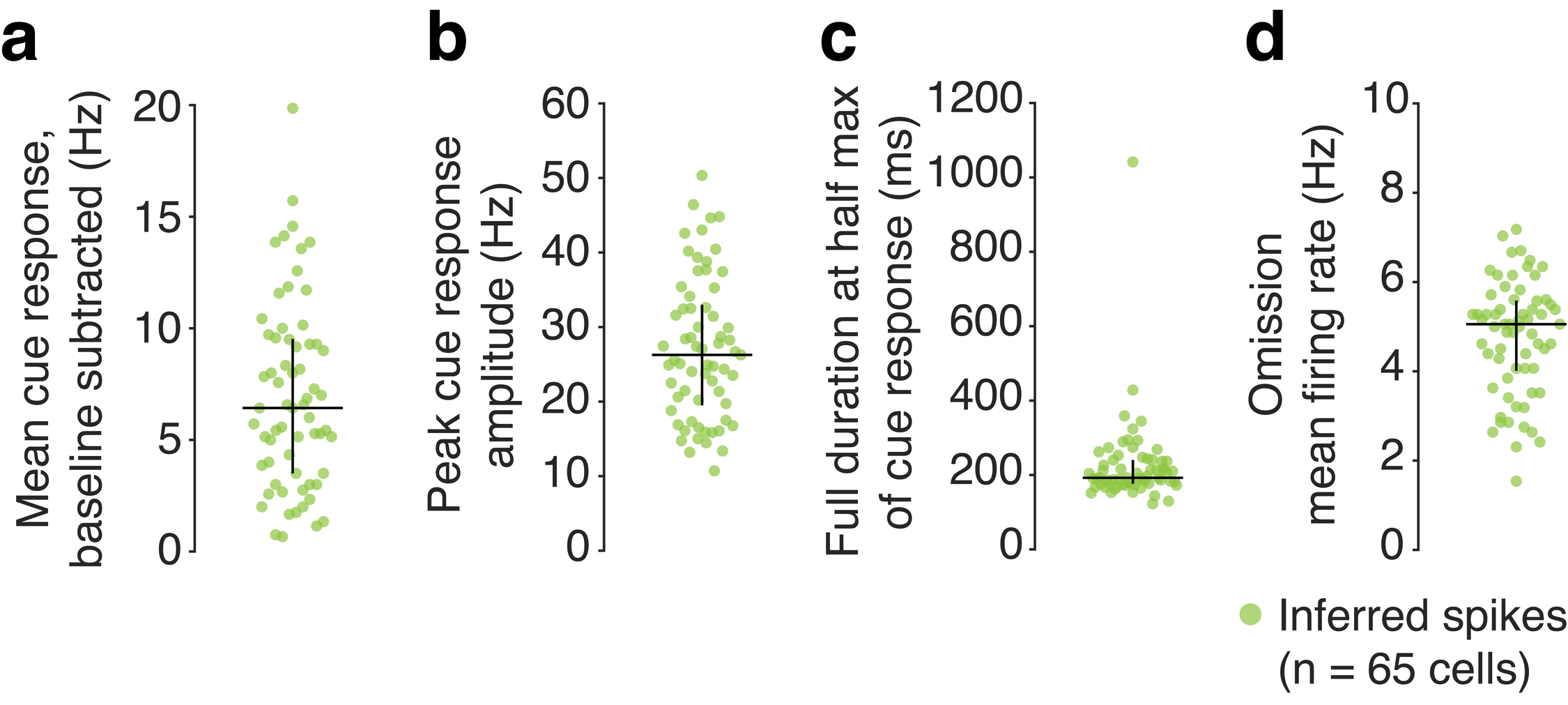

Supplement: S10 Fig — a. Mean cue response during omission trials, where cue response is the mean firing rate over first 500 ms following cue presentation, baseline subtracted using the mean firing rate over a 1 s period before cue (median = 6.4 Hz, Q1 = 3.5 Hz, Q3 = 9.5 Hz). b. Peak cue response amplitude in inferred spikes during omission trials, where peak is maximum value of PSTH in the first 500 ms period following cue presentation (median = 26.3 Hz, Q1 = 19.5 Hz, Q3 = 33.0 Hz). c. Full duration at half max of cue response in inferred spikes during omission trials (median = 192.5 ms, Q1 = 176.7 ms, Q3 = 240.5 ms). d. Mean firing rate in inferred spikes over 1,300 ms period following onset of reward omission (median = 5.1 Hz, Q1 = 4.0 Hz, Q3 = 5.6 Hz). Vertical bars are interquartile range (Q1 and Q3). (TIF) [file pone.0252345.s010.tif]

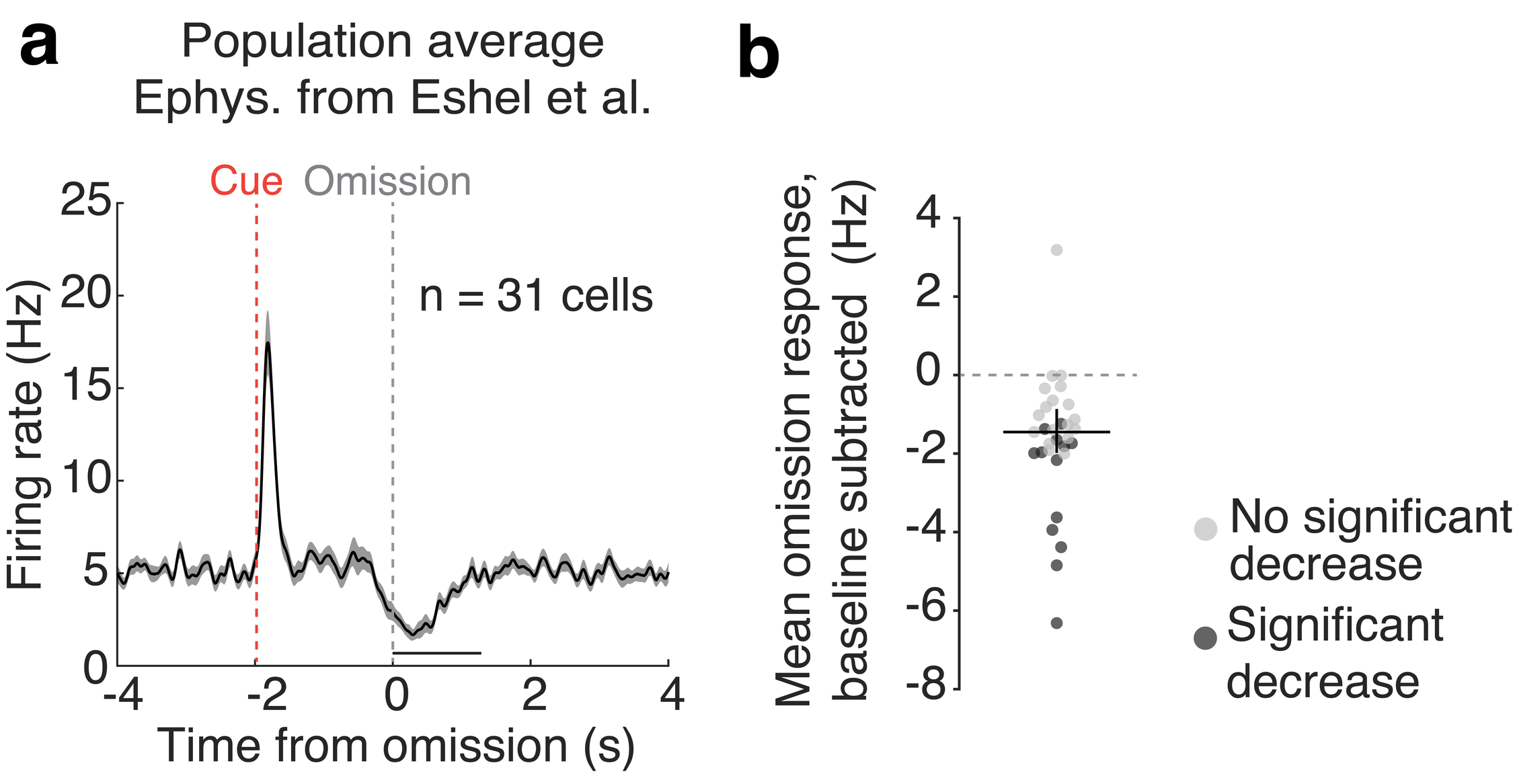

Supplement: S11 Fig — a. Mean population firing rate from electrophysiology spikes around unexpected omission of reward following presentation reward-predictive odor cue. Shaded areas are SEM. h. Mean omission response of electrophysiology spikes, where omission response is mean firing rate over 1,300 ms following onset of reward omission, baseline subtracted using the mean firing rate over 1 s period before trial start (median = -1.45 Hz, Q1 = -1.98 Hz, Q3 = -0.87 Hz). Neurons that exhibited a significant decrease in firing following reward omission (13/31 neurons; 41.9% of population) are darker grey. Vertical bars are interquartile range (Q1 and Q3). Only the first 7 trials for each neuron were used for determination of significant omission response to correspond to the inferred spike data. Note that this data is not used to directly compare to inferred spike modalities because of differences in behavioral paradigms, including differences in reward-predictive cue modality. Data from Eshel et al. [39]. (TIF) [file pone.0252345.s011.tif]

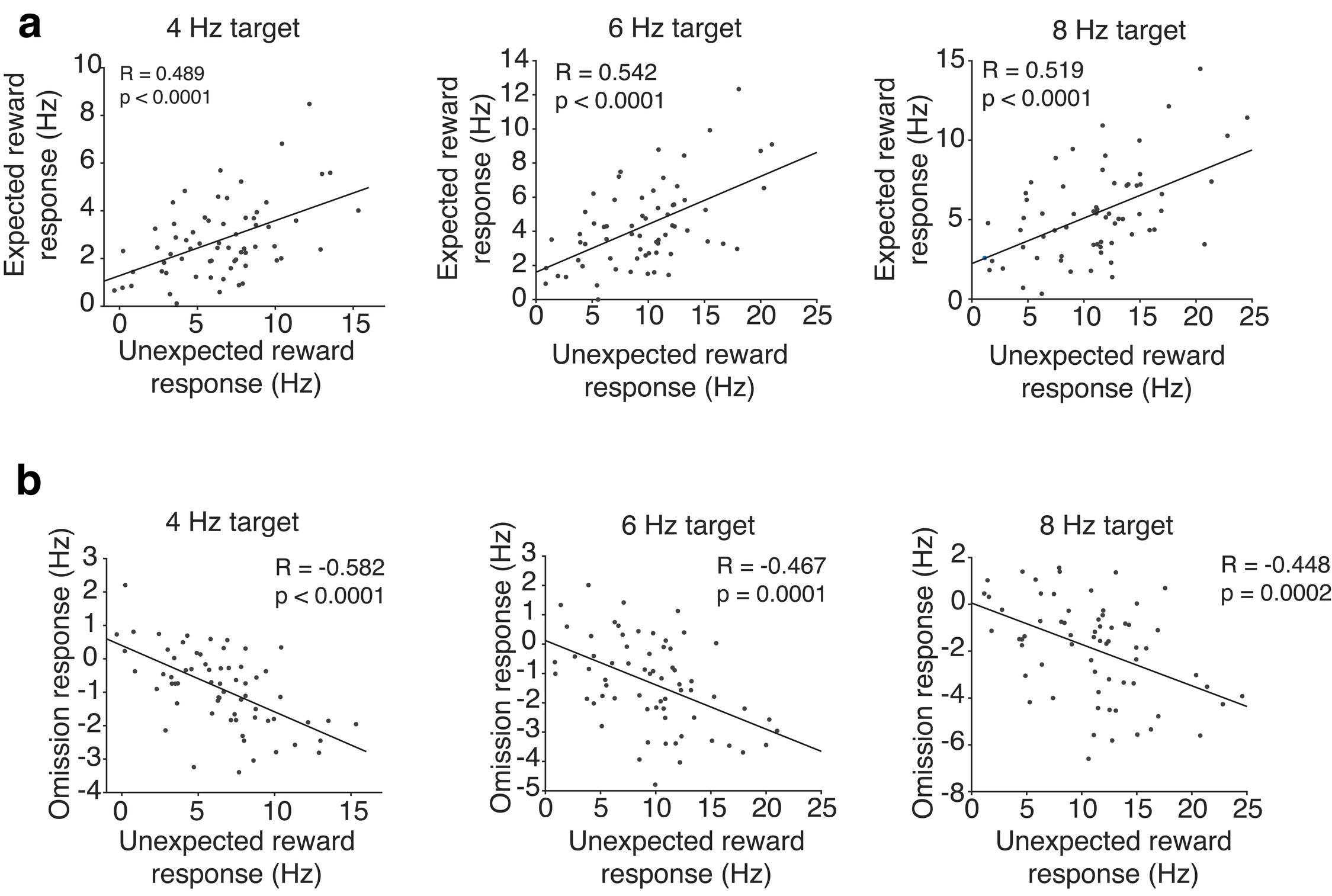

Supplement: S12 Fig — a. Scatterplot of expected reward response versus unexpected reward response recapitulates correlations in Eshel et al. [39] when using tuning parameters for a range of inferred firing rate. b. Scatterplots of omission response versus unexpected reward response recapitulates correlations in Eshel et al. [39] when using tuning parameters for a range of inferred firing rates. Responses in a and b are baseline subtracted. (TIF) [file pone.0252345.s012.tif]

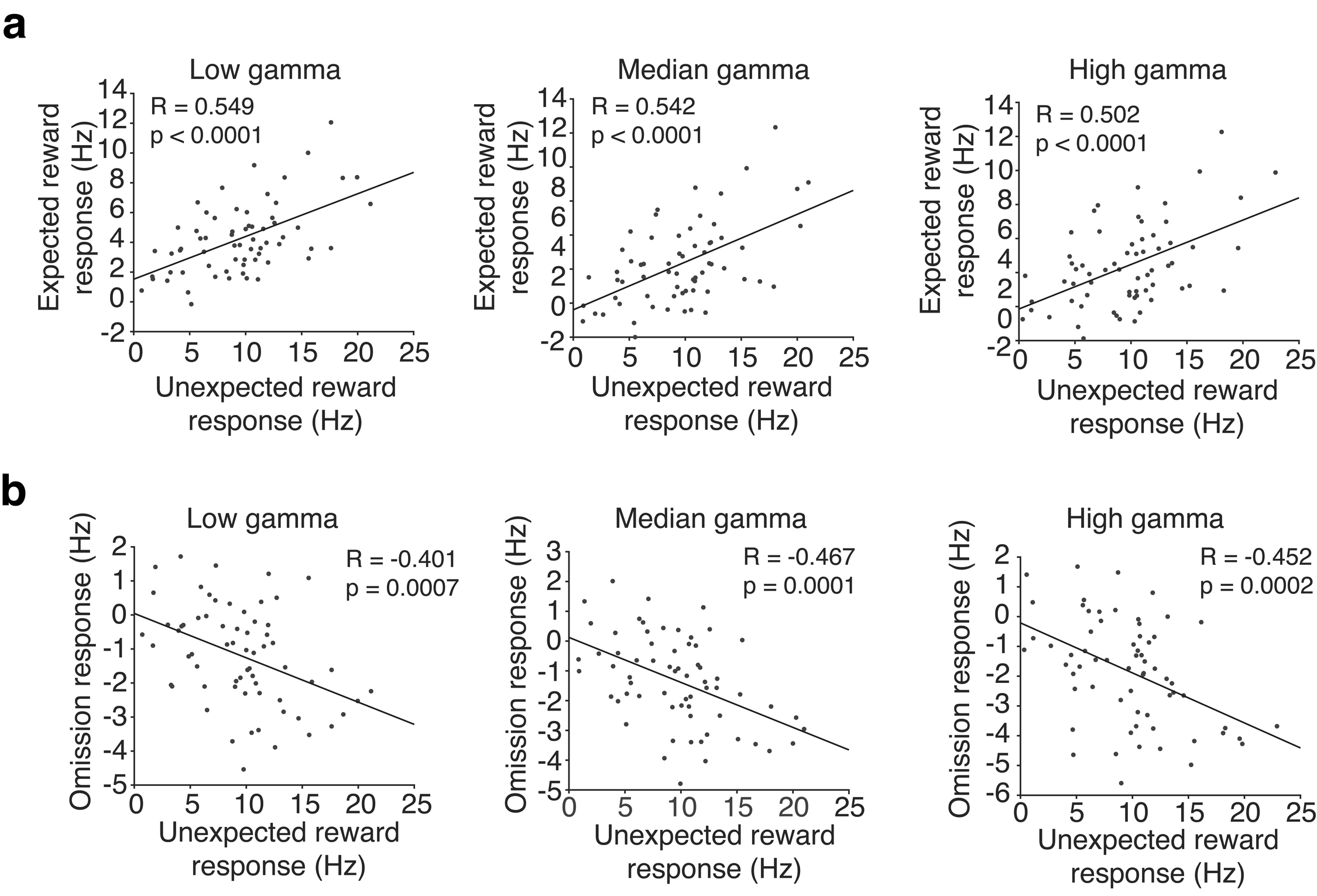

Supplement: S13 Fig — a. Scatterplot of expected reward response versus unexpected reward response recapitulates correlations in Eshel et al. [39] when λ is selected to target a 6 Hz average estimated firing rate and the decay rate γ is selected as the 25th percentile (low gamma), median, or 75th percentile (high gamma) estimated decay rate from the in vitro experiment (Fig 1D). b. Scatterplots of omission response versus unexpected reward response recapitulates correlations in Eshel et al. [39] when using tuning parameters for a range of inferred firing rates. Responses in a and b are baseline subtracted. (TIF) [file pone.0252345.s013.tif]

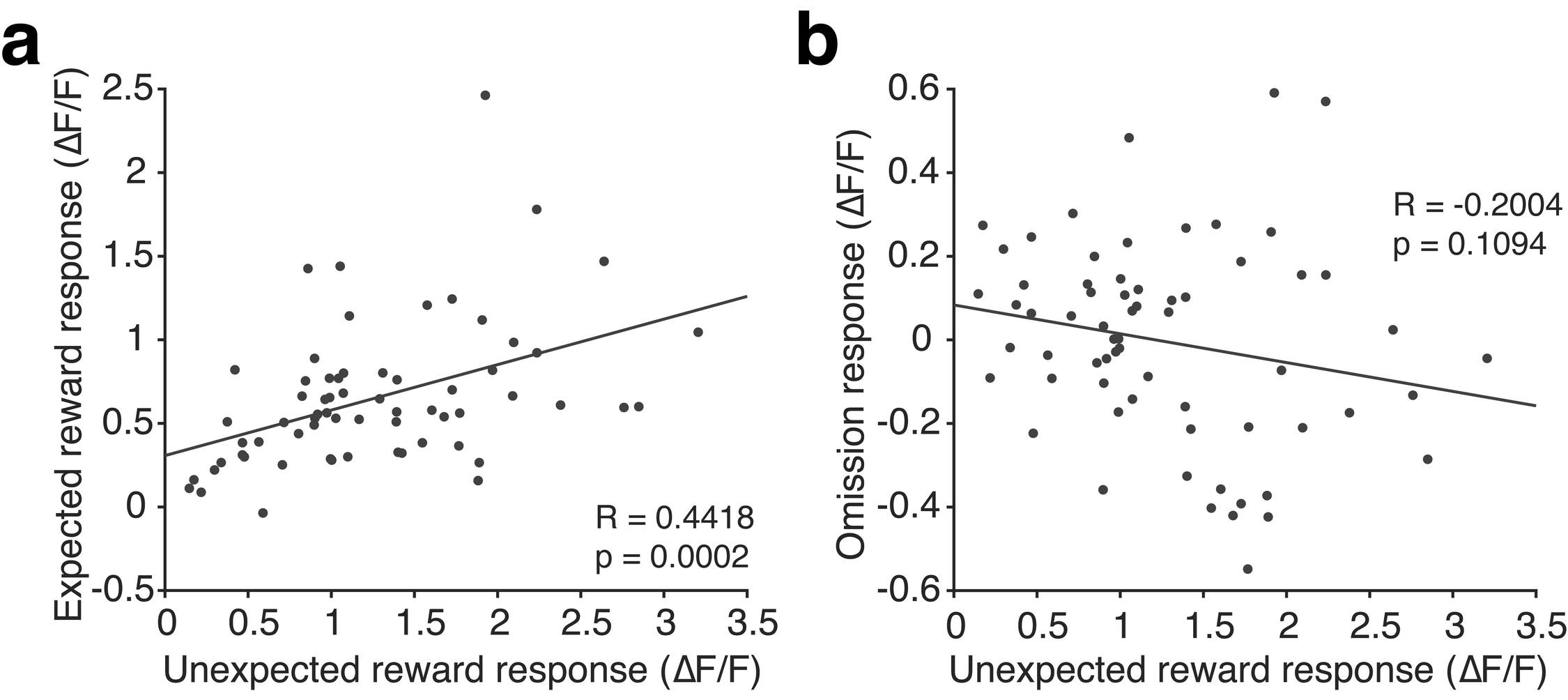

Supplement: S14 Fig — a. Scatterplot of expected reward response versus unexpected reward response. Responses are calculated by finding mean ΔF/F value over a 600 ms window following reward presentation and baseline subtracting the mean ΔF/F value over a 1 s window before trial start (1 s before cue for expected reward; 1 s before reward for unexpected reward). b. Scatterplot of omission response versus unexpected reward response. Omission response is calculated by finding the mean ΔF/F value over a 1,300 ms window following reward omission and baseline subtracting the mean ΔF/F value over a 1 s window before cue. (TIF) [file pone.0252345.s014.tif]

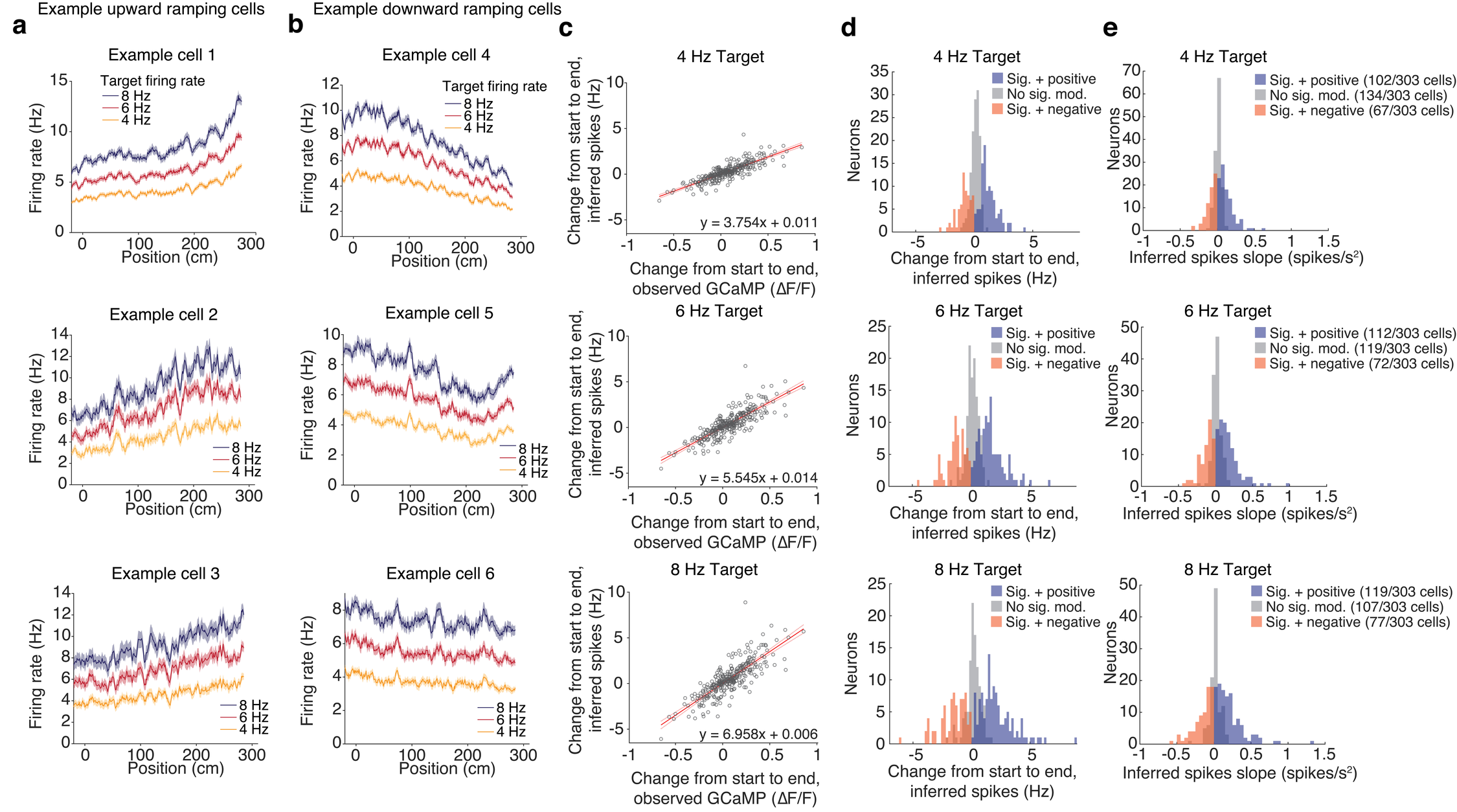

Supplement: S15 Fig — Results of spike inference during the virtual reality T-maze task in Fig 4 when tuning parameters approximating mean firing rates of 4, 6, or 8 Hz are used. a. Mean inferred firing rate by position for example upward-ramping cells over a range of tuning parameters. b. Mean inferred firing rate by position for example downward-ramping cells over a range of tuning parameters. c. For each tuning parameter, scatterplots showing how change in observed GCaMP from beginning to end of maze for each neuron relates to change in inferred firing rate for that neuron. Each data point represents a single neuron and its mean change in observed GCaMP and inferred firing rate. Red line is linear least-squares fit; dotted region is 95% confidence of the fit. d. For each tuning parameter, histograms of changes of inferred firing rate for each neuron, color coded by whether a neuron’s probability of firing significantly decreased (red), increased (blue), or did not change (grey) with increases in maze position. e. For each tuning parameter, histograms of changes of the inferred firing rate slope for each neuron, color coded by whether a neuron’s probability of firing significantly decreased (red; mean slope = -0.09 spikes/s2, n = 67/303 neurons for 4 Hz target; mean slope = -0.12 spikes/s2, n = 72/303 neurons for 6 Hz target; mean slope = -0.15 spikes/s2, n = 77/303 neurons for 8 Hz target), increased (blue; mean slope = 0.13 spikes/s2, n = 102/303 neurons for 4 Hz target; mean slope = 0.18 spikes/s2; n = 112/303 neurons for 6 Hz target; mean slope = 0.22 spikes/s2, n = 119/303 neurons for 8 Hz target), or did not change (grey; mean slope = 0.01 spikes/s2, n = 134/303 neurons for 4 Hz target; mean slope = 0.02 spikes/s2; n = 119/303 neurons for 6 Hz target; mean slope = 0.02 spikes/s2, n = 107/303 neurons for 8 Hz target) with increases in maze position. Significant position modulation determined by a generalized linear model (GLM), where the inferred spikes were predicted by mouse pos [file pone.0252345.s015.tif]

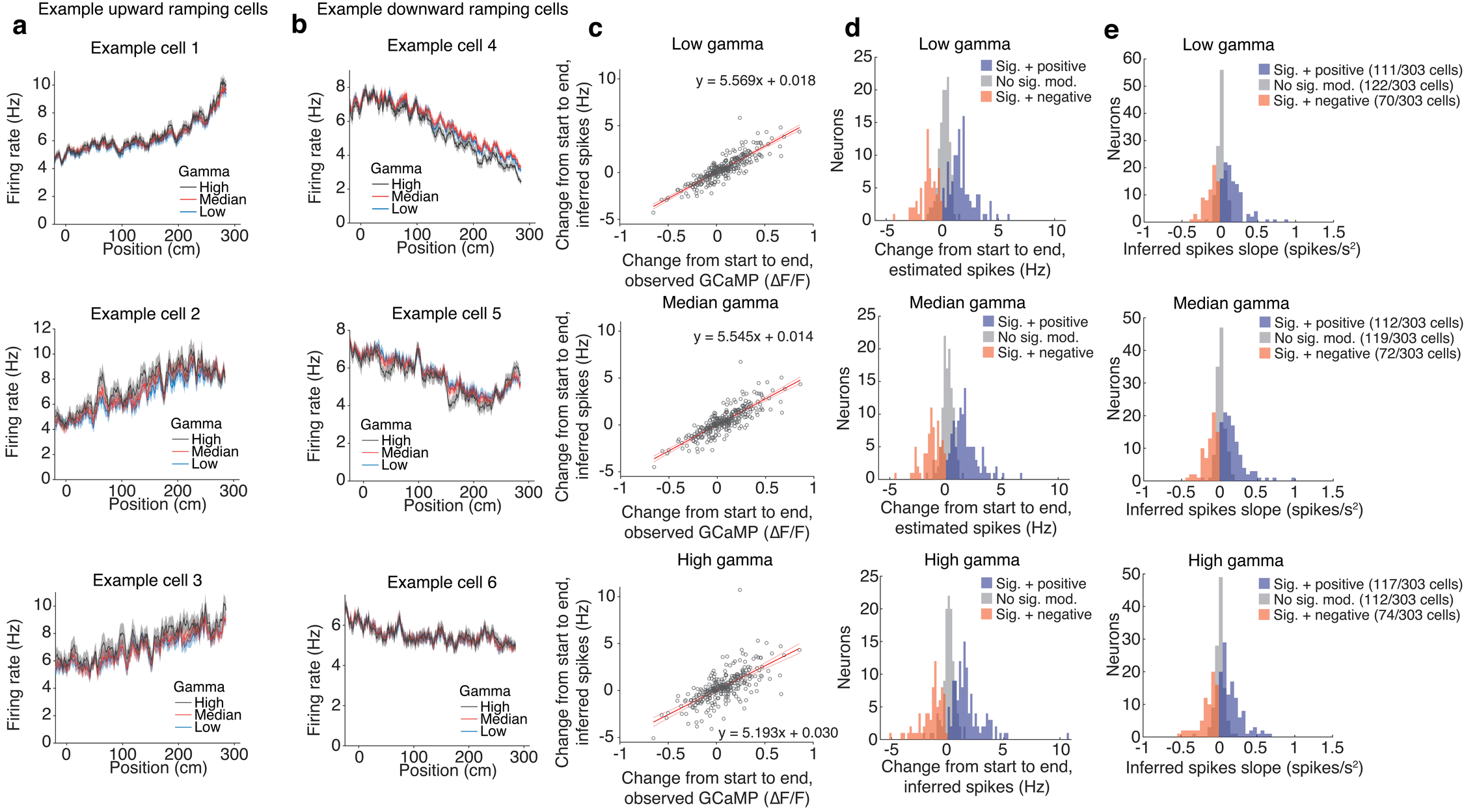

Supplement: S16 Fig — Results of spike inference during the virtual reality T-maze task in Fig 4 when λ is selected to target a 6 Hz average estimated firing rate and the decay rate γ is selected as the 25th percentile (low gamma), median, or 75th percentile (high gamma) estimated decay rate from the in vitro experiment (Fig 1D). a. Mean inferred firing rate by position for example upward-ramping cells over a range of decay rates. b. Mean inferred firing rate by position for example downward-ramping cells over a range of decay rates. c. For each decay rate, scatterplots showing how change in observed GCaMP from beginning to end of maze for each neuron relates to change in inferred firing rate for that neuron. Each data point represents a single neuron and its mean change in observed GCaMP and inferred firing rate. Red line is linear least-squares fit; dotted region is 95% confidence of the fit. d. For each decay rate, histograms of changes of inferred firing rate for each neuron, color coded by whether a neuron’s probability of firing significantly decreased (red), increased (blue), or did not change (grey) with increases in maze position. e. For each decay rate, histograms of changes of the inferred firing rate slope for each neuron, color coded by whether a neuron’s probability of firing significantly decreased (red; mean slope = -0.12 spikes/s2, n = 70/303 neurons for low gamma; mean slope = -0.12 spikes/s2, n = 72/303 neurons for median gamma; mean slope = -0.14 spikes/s2, n = 74/303 neurons for high gamma), increased (blue; mean slope = 0.18 spikes/s2, n = 111/303 neurons for low gamma; mean slope = 0.18 spikes/s2; n = 112/303 neurons for median gamma; mean slope = 0.18 spikes/s2, n = 117/303 neurons for high gamma), or did not change (grey; mean slope = 0.02 spikes/s2, n = 122/303 neurons for low gamma; mean slope = 0.02 spikes/s2; n = 119/303 neurons for median gamma; mean slope = 0.01 spikes/s2, n = 112/303 neurons for high gamma) with increases in maze position. Significant posi [file pone.0252345.s016.tif]
