# Supplementary material for: Inferring spikes from calcium imaging in dopamine neurons
Source: PLoS One. 2021 Jun 4;16(6):e0252345. doi: 10.1371/journal.pone.0252345 (PMC8177503; doi:10.1371/journal.pone.0252345)
Supplement: Attachment — Submitted filename: PLOSOneReview_Fleming.docx [file pone.0252345.s017.docx]

Fleming et al present important empirical and modeling results aimed at inferring spiking from calcium signals in dopaminergic neurons. Although the scope and applicability is somewhat limited, the results will be relevant to the increasing number of labs using calcium imaging approaches to studying *in-vivo* dynamics in this cell-type.

Although the authors acknowledge that the decay and rate parameters vary across neurons (and therefore the main utility of the model is in interpreting population level dynamics rather than single neurons), it is unclear from the data presented how much the parameters actually vary across neurons and how small changes in the parameter estimates might impact inferred spiking across the population. This point seems critical for determining whether the parameter estimates given can be broadly applied to dopamine neurons *in-vivo*, especially given the significant variability observed in other regions and cell-types. Specifically:

1. Decay parameter medians and ranges for *in-vitro* data are shown in Figure 1, but the authors should include data points for each individual neuron, especially given that the number of neurons for each condition is quite low (n’s should be listed in the legend).
2. Each ‘recording’ in Figure 1 was considered an independent measurement even though two recordings were made for some neurons (lines 59-61). It’s not clear whether separate recordings from the same neurons were used to calculate the median decay times used for the *in-vivo* inference.
3. It would be useful to determine how varying the rate and decay across neurons within some range (ideally estimated from *in-vitro* recordings), impacts estimates of phasic increases and decreases and slow ramps. Perhaps small changes would not change the population spiking estimates much, but this seems important to know when applying to future *in-vivo* recordings which may sample from different dopamine neuron populations.

In Figure 4, the authors show that positive and negative ramping spike rates can be recovered from ramping calcium signals in single neurons using their model. However, nothing is shown to validate their inference against electrophysiological data as in Figures 2 and 3. Does their spike inference model reveal features of the ramping that cannot be gleaned from the calcium signal alone (as for reward omission trials)? Other studies (i.e. Wei et al 2020) have argued that negative and positive ramps in single neuron spiking are differentially represented in calcium signaling – does this model reconcile those discrepancies? Since the model parameters can vary across single neurons, can the authors show features of spike ramping at the population level that can be accurately recovered from their model (e.g. rates or proportions of positive and negative ramping neurons?)

Other minor points:

1. To account for the rise time kinetics of the indicator, the authors state that they shift the inferred spike times by 4 time steps: some discussion of whether this shift is consistent among the indicators or neurons might be useful, along with a discussion on how this shift might be tuned, and why it is optimal to consider this potential third free parameter in this way rather than build in a double exponential with a rise parameter, as is often used to model these calcium transients
2. The type of GCaMP (m or f) used for imaging experiments should be indicated in each figure legend if possible
3. More discussion of the applications and limitations of this model to future *in-vivo* dopamine neuron recordings at the single neuron and population levels would be beneficial for potential adopters.
4. Do model parameters differ across different midbrain regions? The *in-vitro* data may be too limited to address this point, but some discussion may be pertinent.
