# Supplementary material for: Inferring spikes from calcium imaging in dopamine neurons
Source: PLoS One. 2021 Jun 4;16(6):e0252345. doi: 10.1371/journal.pone.0252345 (PMC8177503; doi:10.1371/journal.pone.0252345)
Supplement: Attachment — Submitted filename: Reviewer response.docx [file pone.0252345.s018.docx]

Main Comments

**1. The authors do a good job of explaining their parameter estimation procedures from in vitro data, but we note that there is considerable variability in the decay rate, especially for the 6f 37C condition, where they have only 5 cells (9 recordings). That variability presents several issues. First, if the larger variability seen in that case (Fig. 1d) is real, it could be problematic for accurately estimating spiking for VTA DA neurons for this particularly common experimental condition, especially using just a single set of validated parameters (e.g. tau=1 sec, lambda=6 Hz). Second, the variability masks the ability to state whether the systematic trends in Fig. 1d between the different calcium sensors at different temperatures are significant. One would perhaps expect that the body temperature decay per time step is faster (smaller value) than for 30C, and that the 6f sensor would be faster than the 6m sensor at the same temperature. Yet the authors are not able to conclude that given the variability. We thus recommend doubling the number of cells, so it is comparable to that of the other two conditions.**

Thank you for the suggestion. We agree that the variability in decay rate<https://www.codecogs.com/eqnedit.php?latex=%5Cgamma#0> observed in the GCaMP6f 37C condition raised the possibility that the relatively small sample size may have resulted in a poor estimate of the decay rate. We have now more than doubled the total number of cells (n = 12) for the GCaMP6f 37C condition (see **Fig. 1d,e** pasted here). The median decay barely changed, and as expected, the 6m 37C decay rate is slower than the 6f 37C decay rate (p = 0.005; two-sample t-test), and the 6f 30C decay rate is slower than the 6f 37C decay rate (p = 0.008; two-sample t-test).

**Excerpt from Fig 1: Estimated decay rates from in vitro experiment. d.** Estimated decay rate per timestep at 66.66 Hz for GCaMP6f at 30°C (median = 0.987, Q1 = 0.983, Q3 = 0.988), GCaMP6f at 37°C (median = 0.975, Q1 = 0.971, Q3 = 0.980), and GCaMP6m at 37°C (median = 0.986, Q1 = 0.983, Q3 = 0.991). **e.** Decay time (half life, t_1/2_) for GCaMP6f at 30°C (median = 0.772 s, Q1 = 0.619 s, Q3 = 0.866 s), GCaMP6f at 37°C (median = 0.412 s, Q1 = 0.359 s, Q3 = 0.523 s), and GCaMP6m at 37°C (median = 0.725 s, Q1 = 0.614 s, Q3 = 1.100 s).

**2. Related to the above, we wonder how much error is introduced by the variation in the decay per time step when only the median or mean value of the (small) population is used for all cells. For example, might there actually be two different subpopulations of DA neurons (e.g. tonic and bursty), so that the median/mean does not do well for one or the other? Consistent with that, Fig. 1f (middle) seems to show some particularly large spike estimation errors for the 6f/37C case, even when the firing rate is matched to the electrophysiologically observed rate. The cost of misestimating the decay rate would be helpful to understand, and the authors should generate a similar set of curves as Fig. 1f except with the decay rate along the x-axis.**

We apologize for being unclear. *In vivo*, all dopamine neurons are thought to display phasic bursts, as well as tonic activity. Consistent with this, in the *in vivo* DA electrophysiology data set, all neurons exhibit burst activity (at least 10% of spikes occur as a series of 3 or more spikes with an interspike interval of 100 ms; **Supp. fig. 1a**) and also display tonic firing during the periods without cues or rewards (**Fig. 2j**). In contrast, and as previously reported [(1,2)](https://paperpile.com/c/BQqrn1/D39s+DTgX), there is a subset of DA neurons that display pacemaker activity but do not exhibit phasic activity *in vitro*. Due to the lack of phasic activity, we consider these cells a worse approximation for the *in vivo* data, and therefore to determine median decay rates we only consider *in vitro* cells that have some phasic activity (**Supp. fig. 1**; this rationale is also detailed in the ***In vitro* data organization** subsection of the **Methods** in the manuscript).

That being said, we do not think our results are highly dependent on the exact choice of decay rate. To clarify this, based on your helpful suggestion, we have created graphs similar to those in **Fig. 1f** that display the quality of estimation as a function of the decay rate. We show that, in general, the spike estimates are quite accurate over a range of decay rates, and in fact the difference between the decay rates for pacemaker vs bursting DA neurons as estimated *in vitro* (for 6f 37C, pacemaker median decay rate = 0.969, bursting cell decay rate = 0.975; for 6m 37C, pacemaker decay = 0.986, bursting cell decay rate = 0.986) should not substantially impact the quality of our estimates. These graphs are reproduced below for your convenience, and also appear as **Fig. 1g** in the revised manuscript.

**Excerpt from Fig 1g: van Rossum distance as a function of decay rate.** van Rossum distance for each *in vitro* experimental condition and for each recording as a function of the decay rate<https://www.codecogs.com/eqnedit.php?latex=%5Cgamma#0> when the tuning parameter matches the observed firing rate of the recording. The shaded region and vertical dark grey line represent the estimated interquartile ranges and median values of the decay rate in Fig 1d.

Lastly, we note the reviewer’s concern that there are “some particularly large spike estimation errors for the 6f/37C case, even when the firing rate is matched to the electrophysiologically observed rate” in **Fig. 1f**. These recordings also show large van Rossum distances in our new decay rate plots (above). In fact, these two recordings with particularly large errors belong to the same cell, and by eye appear to be attributable to poor correspondence between observed spikes and GCaMP fluorescence. Below, we include a detail of a trace from one of these recordings, where, for example, the first spike burst precedes an increase in fluorescence by an unusually long time lag, and the second spike burst appears to not correspond to a large increase in observed fluorescence.

**Example of recording with poor correspondence between observed spikes and observed fluorescence.** Detail of a trace where spike inference performs poorly, presumably because of poor correspondence between observed spikes and observed fluorescence. Spike bursts do not show consistent onset of fluorescence rise (compare first burst to penultimate burst), and do not always correspond to a large increase in fluorescence (see second burst). This detail is not included in the manuscript as its own figure. An example trace from this recording can be found in **Supplementary figure 4** of the manuscript.

Because we could not identify a clear experimental cause for the discrepancy between observed fluorescence and observed spikes, we did not exclude this data. However, we believe that this is anomalous and attributable to experimental error, and certainly it is not a result of poor performance by our spike inference model (since we do not expect spike inference to perform well in a setting where, by eye, there is very poor correspondence between observed spikes and observed fluorescence).

**3. For several neurons/recordings, the estimation tends to predict higher calcium levels than observed in the troughs between bursts – never lower. Can the authors comment on why there is such a systematic error, and perhaps a discussion on what could be done to improve the model (or further caveats on the current model’s limitations)?**

Thanks for pointing this out.

First of all, your comment caused us to revisit the code for **Fig. 1c**, which turned out to have been calculated incorrectly, which made it look like the estimated calcium was always larger than the observed fluorescence. We have corrected this mistake in the revised manuscript. This mistake was specific to the code used to create the schematic for **Fig. 1c** and did not affect any analysis elsewhere.

Putting aside this issue with **Fig. 1c**, you raise a good point about the fact that we can see --- for instance, from **Figs. 1h,j** --- that the model is misspecified. In particular, even though the estimated spikes are very close to the observed spikes in these three panels, the estimated calcium does not perfectly match up with the true spikes: we can see this very clearly in **Figs. 1h,j**. There are at least three reasons for this:

1) The second line in the model

<https://www.codecogs.com/eqnedit.php?latex=y_%7Bt%7D%20%3D%20c_%7Bt%7D%20%2B%20%5Cepsilon_%7Bt%7D%2C%20%5Cquad%20%5Cepsilon_%7Bt%7D%20%5Coverset%7B%5Ctext%7Biid%7D%7D%7B%5Csim%7D%20%5Cmathcal%7BN%7D(0%2C%20%5Csigma%5E%7B2%7D)%2C%20%5Cquad%20t%20%3D%201%2C%20%5Cldots%2C%20T%2C#1>

<https://www.codecogs.com/eqnedit.php?latex=c_%7Bt%7D%20%3D%20%5Cgamma%20c_%7Bt-1%7D%20%2B%20z_%7Bt%7D%2C%20%5Cquad%20t%20%3D%202%2C%20%5Cldots%2C%20T%20%5Cnonumber%2C#1>

is an overly simplistic representation of the calcium dynamics in response to a spike. In particular, this model says that if there is a spike at the *t*th timepoint, then the calcium increases instantaneously before continuing its exponential decay. However, in reality, the calcium (or, at least, the observed fluorescence) increases slowly in response to a spike, over the course of several timesteps.

2) The first line in the model above assumes that the T error terms (one per timepoint) are independent. In reality, they are likely to be at least somewhat correlated.
3) As pointed out in one of the reviewer’s earlier comments, we estimate a single value of<https://www.codecogs.com/eqnedit.php?latex=%5Cgamma#0> across all recordings, for a given temperature and GCaMP sensor. While this yields adequate results, and while the overall quality of spike estimation is not compromised (see e.g. **Fig. 1g** in the revised manuscript), it is certainly the case that the value of<https://www.codecogs.com/eqnedit.php?latex=%5Cgamma#0> used is not exactly right.

These three sources of model misspecification contribute to the fact that the estimated calcium does not provide a perfect fit to the observed fluorescence. Nonetheless, our model provides quite good results. While it would be possible to consider a model that avoids either of these three sources of misspecification --- for instance, by fitting a second-order rather than a first-order auto-regressive model to allow for a slow increase in calcium over time; by allowing for correlation among the error terms; or by estimating a separate<https://www.codecogs.com/eqnedit.php?latex=%5Cgamma#0> for each recording --- we have not found these avenues to be fruitful. In general, fitting a more complex model may lead to a small reduction in bias at the expense of a potentially large increase in variance; we have found that the current model, though a bit simplistic, positions us well in terms of the bias-variance trade-off.

**4. Although Data is fully available without restrictions, there is no indication of where to obtain the data. According to PLoS requirements, availability upon request is insufficient and some information on where the data can be found (e.g. repository) is recommended.**

We will make all of the data obtainable on our Github site.

**5. Although the authors acknowledge that the decay and rate parameters vary across neurons (and therefore the main utility of the model is in interpreting population level dynamics rather than single neurons), it is unclear from the data presented how much the parameters actually vary across neurons and how small changes in the parameter estimates might impact inferred spiking across the population. This point seems critical for determining whether the parameter estimates given can be broadly applied to dopamine neurons in-vivo, especially given the significant variability observed in other regions and cell-types. Specifically:**

**5.1. Decay parameter medians and ranges for in-vitro data are shown in Figure 1, but the authors should include data points for each individual neuron, especially given that the number of neurons for each condition is quite low (n’s should be listed in the legend).**

Thank you for this suggestion. We have updated the way this data is displayed and now display data points for each individual neuron. In the case where we had multiple recordings from the same cell, we calculated average decay rates across those recordings to get a single decay rate for each neuron. Please see **Fig. 1d-e** in the revised manuscript.

**5.2. Each ‘recording’ in Figure 1 was considered an independent measurement even though two recordings were made for some neurons (lines 59-61). It’s not clear whether separate recordings from the same neurons were used to calculate the median decay times used for the in-vivo inference.**

Thank you - we think this is a good point. Per this suggestion, we have updated our calculation of median decay times to be the median over cells, not recordings. In cases where cells had multiple recordings, the estimated decay from that cell was calculated by taking the mean of estimated decays from the individual recordings. We have updated the **Methods** accordingly with clarifying descriptions in both the **Estimating the decay rate**<http://www.sciweavers.org/tex2img.php?bc=Transparent&fc=Black&im=jpg&fs=100&ff=modern&edit=0&eq=%5Cgamma#0>subsection of the ***In vitro* data analysis** section and the **Decay rate determination** subsection of the ***In vivo* data analysis** section.

**5.3. It would be useful to determine how varying the rate and decay across neurons within some range (ideally estimated from in-vitro recordings), impacts estimates of phasic increases and decreases and slow ramps. Perhaps small changes would not change the population spiking estimates much, but this seems important to know when applying to future in-vivo recordings which may sample from different dopamine neuron populations.**

Thank you for this important suggestion. We show now the results of estimations on our Pavlovian data when the tuning rate is chosen at three firing rates, all in the known range of tonic DA neuron spiking activity (4, 6, and 8 Hz).

**Supplementary figure 7. Tuning rate comparison for Pavlovian experiment**. **a.** Mean population firing rates from inferred spikes around presentation of unexpected reward when<http://www.sciweavers.org/tex2img.php?bc=Transparent&fc=Black&im=jpg&fs=100&ff=modern&edit=0&eq=%5Clambda#0> is selected to target a 4, 6, or 8 Hz average estimated firing rate (n = 65 cells). **b-e.** Comparison of inferred spikes over a range of<http://www.sciweavers.org/tex2img.php?bc=Transparent&fc=Black&im=jpg&fs=100&ff=modern&edit=0&eq=%5Clambda#0> values and electrophysiology spikes from Eshel et al., 2016. **b.** Unexpected reward response, where reward response is the mean firing rate over the first 600 ms following reward presentation, baseline subtracted using the mean firing rate over a 1 s period before reward presentation (inferred spikes over baseline median = 6.5 Hz, Q1 = 3.9 Hz, Q3 = 8.2 Hz for<http://www.sciweavers.org/tex2img.php?bc=Transparent&fc=Black&im=jpg&fs=100&ff=modern&edit=0&eq=%5Clambda#0>target 4 Hz; median = 9.8 Hz, Q1 = 6.2 Hz, Q3 = 12.2 Hz, for<http://www.sciweavers.org/tex2img.php?bc=Transparent&fc=Black&im=jpg&fs=100&ff=modern&edit=0&eq=%5Clambda#0>target 6 Hz; median = 11.5 Hz, Q1 = 7.1 Hz, Q3 = 14.0 Hz, for<http://www.sciweavers.org/tex2img.php?bc=Transparent&fc=Black&im=jpg&fs=100&ff=modern&edit=0&eq=%5Clambda#0>target 8 Hz; electrophysiology spikes over baseline median = 9.5 Hz, Q1 = 6.3 Hz, Q3 = 12.0 Hz). **c.** Peak reward response amplitude in inferred and electrophysiology spikes, where peak is maximum value of PSTH in the first 600 ms period following reward presentation (inferred spikes median = 19.7 Hz, Q1 = 15.0 Hz, Q3 = 27.2 Hz, for<http://www.sciweavers.org/tex2img.php?bc=Transparent&fc=Black&im=jpg&fs=100&ff=modern&edit=0&eq=%5Clambda#0>target 4 Hz; median = 29.0 Hz, Q1 = 23.7 Hz, Q3 = 38.6 Hz, for<http://www.sciweavers.org/tex2img.php?bc=Transparent&fc=Black&im=jpg&fs=100&ff=modern&edit=0&eq=%5Clambda#0>target 6 Hz; median = 34.4 Hz, Q1 = 28.1 Hz, Q3 = 44.3 Hz, for<http://www.sciweavers.org/tex2img.php?bc=Transparent&fc=Black&im=jpg&fs=100&ff=modern&edit=0&eq=%5Clambda#0>target 8 Hz; electrophysiology median = 30.5 Hz, Q1 = 22.6 Hz, Q3 = 40.9 Hz). **d.** Full duration at half max of reward response peak in inferred and electrophysiology spikes (inferred spikes median full duration at half max = 173.4 ms, Q1 = 156.8 ms, Q3 = 226.8 ms for<http://www.sciweavers.org/tex2img.php?bc=Transparent&fc=Black&im=jpg&fs=100&ff=modern&edit=0&eq=%5Clambda#0>target 4 Hz; median = 183.9 ms, Q1 = 163.6 ms, Q3 = 235.9 ms for<http://www.sciweavers.org/tex2img.php?bc=Transparent&fc=Black&im=jpg&fs=100&ff=modern&edit=0&eq=%5Clambda#0>target 6 Hz; median = 196.2 ms, Q1 = 175.8 ms, Q3 = 242.0 ms for<http://www.sciweavers.org/tex2img.php?bc=Transparent&fc=Black&im=jpg&fs=100&ff=modern&edit=0&eq=%5Clambda#0>target 8 Hz; electrophysiology median full duration at half max = 153.5 ms, Q1 = 120.4 ms, Q3 = 272.5 ms). **e.** Mean tonic firing rates prior to presentations of unexpected reward, where tonic firing rates are calculated as the mean firing rate over a 1 s period prior to reward presentation (inferred spikes tonic firing rate median = 3.9 Hz, Q1 = 3.4 Hz, Q3 = 4.1 Hz, for<http://www.sciweavers.org/tex2img.php?bc=Transparent&fc=Black&im=jpg&fs=100&ff=modern&edit=0&eq=%5Clambda#0>target 4 Hz; median = 5.7 Hz, Q1 = 5.3 Hz, Q3 = 6.0 Hz, for<http://www.sciweavers.org/tex2img.php?bc=Transparent&fc=Black&im=jpg&fs=100&ff=modern&edit=0&eq=%5Clambda#0>target 6 Hz; median = 7.6 Hz, Q1 = 7.0 Hz, Q3 = 8.3 Hz, for<http://www.sciweavers.org/tex2img.php?bc=Transparent&fc=Black&im=jpg&fs=100&ff=modern&edit=0&eq=%5Clambda#0>target 8 Hz; electrophysiology tonic firing rate median = 5.6 Hz, Q1 = 4.3 Hz, Q3 = 7.5 Hz). Vertical bars are interquartile range (Q1 and Q3). **f.** Mean inferred spikes from population around presentation of expected reward. **g.** Full duration at half max of cue response in inferred spikes (median = 182.3 ms, Q1 = 168.4 ms, Q3 = 203.2 ms for<http://www.sciweavers.org/tex2img.php?bc=Transparent&fc=Black&im=jpg&fs=100&ff=modern&edit=0&eq=%5Clambda#0>target 4 Hz; median = 189.3 ms, Q1 = 168.3 ms, Q3 = 205.6 ms for<http://www.sciweavers.org/tex2img.php?bc=Transparent&fc=Black&im=jpg&fs=100&ff=modern&edit=0&eq=%5Clambda#0>target 6 Hz; median = 197.2 ms, Q1 = 174.9 ms, Q3 = 220.5 ms for<http://www.sciweavers.org/tex2img.php?bc=Transparent&fc=Black&im=jpg&fs=100&ff=modern&edit=0&eq=%5Clambda#0>target 8 Hz). Vertical bars are interquartile range (Q1 and Q3). **h.** Mean population firing rate from inferred spikes around unexpected omission of reward. Shaded areas are SEM. **i**. Mean omission response of inferred spikes, where omission response is mean firing rate over 1,300 ms following onset of reward omission, baseline subtracted using the mean firing rate over 1 s period before trial start (median = -0.7 Hz, Q1 = -1.8 Hz, Q3 = -0.1 Hz, for<http://www.sciweavers.org/tex2img.php?bc=Transparent&fc=Black&im=jpg&fs=100&ff=modern&edit=0&eq=%5Clambda#0>target 4 Hz; median = -1.3 Hz, Q1 = -2.3 Hz, Q3 = -0.1 Hz, for<http://www.sciweavers.org/tex2img.php?bc=Transparent&fc=Black&im=jpg&fs=100&ff=modern&edit=0&eq=%5Clambda#0>target 6 Hz; median = -1.5 Hz, Q1 = -3.4 Hz, Q3 = -0.4 Hz, for<http://www.sciweavers.org/tex2img.php?bc=Transparent&fc=Black&im=jpg&fs=100&ff=modern&edit=0&eq=%5Clambda#0>target 8 Hz). Neurons that exhibited a significant decrease in firing following reward omission (21/65 neurons, 32.3% of population for<http://www.sciweavers.org/tex2img.php?bc=Transparent&fc=Black&im=jpg&fs=100&ff=modern&edit=0&eq=%5Clambda#0>target 4 Hz; 22/65 neurons, 33.6% of population for<http://www.sciweavers.org/tex2img.php?bc=Transparent&fc=Black&im=jpg&fs=100&ff=modern&edit=0&eq=%5Clambda#0>target 6 Hz; 21/65 neurons, 32.3% of population for<http://www.sciweavers.org/tex2img.php?bc=Transparent&fc=Black&im=jpg&fs=100&ff=modern&edit=0&eq=%5Clambda#0>target 8 Hz) are darker green. Vertical bars are interquartile range (Q1 and Q3). All imaging data is of cells expressing GCaMP6f.

As you can see, we observe the same general trends regardless of the target firing rate. In general, the higher the target firing rate, the larger the reward response magnitude (panel **b**), reward peak amplitude (panel **c**), and omission response (panel **i**). The kinetics of the responses appear to also have some dependence on the tuning parameter, where a higher target firing rate generates reward (panel **d**) and reward-predicting cue responses (panel **g**) of slightly longer durations. However, these measures appear quite comparable, with largely overlapping distributions. The most distinct difference observed between tuning parameters is in the tonic firing rate (panel **e**), where the tonic firing rates all appear tightly distributed, with medians just below the respective target firing rates.

We also show the results of estimation when using a range of values for decay rate<http://www.sciweavers.org/tex2img.php?bc=Transparent&fc=Black&im=jpg&fs=100&ff=modern&edit=0&eq=%5Cgamma#0>. For this data, we followed the reviewer recommendation of picking values informed by the *in vitro* experiment and selected the 25th percentile, the median, and 75th percentile decay rate values estimated from the *in vitro* data in **Fig. 1d**.

**Supplementary figure 8. Decay rate comparison for Pavlovian experiment**. **a.** Mean population firing rates (n = 65 cells) from inferred spikes around presentation of unexpected reward when<http://www.sciweavers.org/tex2img.php?bc=Transparent&fc=Black&im=jpg&fs=100&ff=modern&edit=0&eq=%5Clambda#0> is selected to target a 6 Hz average estimated firing rate and the decay rate<http://www.sciweavers.org/tex2img.php?bc=Transparent&fc=Black&im=jpg&fs=100&ff=modern&edit=0&eq=%5Cgamma#0> is selected as the 25th percentile (low gamma), median, or 75th percentile (high gamma) estimated decay rate from the *in vitro* experiment. **b-e.** Comparison of inferred spikes over a range of<http://www.sciweavers.org/tex2img.php?bc=Transparent&fc=Black&im=jpg&fs=100&ff=modern&edit=0&eq=%5Cgamma#0> values and electrophysiology spikes from Eshel et al., 2016. **b.** Unexpected reward response, where reward response is the mean firing rate over the first 600 ms following reward presentation, baseline subtracted using the mean firing rate over a 1 s period before reward presentation (inferred spikes over baseline median = 9.8 Hz, Q1 = 6.2 Hz, Q3 = 12.0 Hz for low gamma; median = 9.8 Hz, Q1 = 6.2 Hz, Q3 = 12.2 Hz, for median gamma; median = 10.3 Hz, Q1 = 6.0 Hz, Q3 = 12.0 Hz, for high gamma; electrophysiology spikes over baseline median = 9.5 Hz, Q1 = 6.3 Hz, Q3 = 12.0 Hz). **c.** Peak reward response amplitude in inferred and electrophysiology spikes, where peak is maximum value of PSTH in the first 600 ms period following reward presentation (inferred spikes median = 27.5 Hz, Q1 = 22.5 Hz, Q3 = 36.9 Hz for low gamma; median = 29.0 Hz, Q1 = 23.7 Hz, Q3 = 38.6 Hz for median gamma; median = 31.3 Hz, Q1 = 24.8 Hz, Q3 = 41.9 Hz for high gamma; electrophysiology median = 30.5 Hz, Q1 = 22.6 Hz, Q3 = 40.9 Hz). **d.** Full duration at half max of reward response peak in inferred and electrophysiology spikes (inferred spikes median full duration at half max = 190.6 ms, Q1 = 164.8 ms, Q3 = 220.3 ms for low gamma; median = 183.9 ms, Q1 = 163.6 ms, Q3 = 235.9 ms for median gamma; median = 187.6 ms, Q1 = 160.5 ms, Q3 = 223.3 ms for high gamma; electrophysiology median full duration at half max = 153.5 ms, Q1 = 120.4 ms, Q3 = 272.5 ms). **e.** Mean tonic firing rates prior to presentations of unexpected reward, where tonic firing rates are calculated as the mean firing rate over a 1 s period prior to reward presentation (inferred spikes tonic firing rate median = 5.6 Hz, Q1 = 5.1 Hz, Q3 = 6.0 Hz, for low gamma; median = 5.7 Hz, Q1 = 5.3 Hz, Q3 = 6.0 Hz for median gamma; median = 5.9 Hz, Q1 = 5.3 Hz, Q3 = 6.1 Hz for high gamma; electrophysiology tonic firing rate median = 5.6 Hz, Q1 = 4.3 Hz, Q3 = 7.5 Hz). Vertical bars are interquartile range (Q1 and Q3). **f.** Mean inferred spikes from population around presentation of expected reward. **g.** Full duration at half max of cue response in inferred spikes (median = 195.1 ms, Q1 = 171.9 ms, Q3 = 209.5 ms for low gamma; median = 189.3 ms, Q1 = 168.3 ms, Q3 = 205.6 ms for median gamma; median = 185.8 ms, Q1 = 165.0 ms, Q3 = 202.3 ms for high gamma). Vertical bars are interquartile range (Q1 and Q3). **h.** Mean population firing rate from inferred spikes around unexpected omission of reward. Shaded areas are SEM. **i**. Mean omission response of inferred spikes, where omission response is mean firing rate over 1,300 ms following onset of reward omission, baseline subtracted using the mean firing rate over 1 s period before trial start (median = -1.1 Hz, Q1 = -2.1 Hz, Q3 = -0.3 Hz, for low gamma; median = -1.3 Hz, Q1 = -2.3 Hz, Q3 = -0.1 Hz, for median gamma; median = -1.7 Hz, Q1 = -3.4 Hz, Q3 = -0.6 Hz, for high gamma). Neurons that exhibited a significant decrease in firing following reward omission (24/65 neurons, 36.9% of population for low gamma; 22/65 neurons, 33.6% of population for median gamma; 25/65 neurons, 38.5% of population for high gamma) are darker green. Vertical bars are interquartile range (Q1 and Q3). All imaging data is from cells expressing GCaMP6f.

As you can see, varying the decay rate appears to have minimal effects on the results. Perhaps the most notable differences are seen in the reward omission response, where higher gamma values appear to generate “pause” responses of slightly larger magnitudes (panel **i**). Nonetheless, it is evident both from the overlap in population raster plots (panels **a,f,h**) that any decay rate selected within the 25th to 75th percentiles of the in vitro experiments will generate highly similar results.

These figures detailing how adjustments to model parameters affects results in the Pavlovian experiments can also be found as **Supp. figs. 7-8** in the revised manuscript.

We also show results of estimations on the VR maze data across this range of tuning parameters (**Supp fig 15**, copied below).

Perhaps most notably, we see in the example PSTHs that, for some cells, the ramp slopes appear to increase in magnitude as the target firing rate increases (Example cell 4 in **Supp fig 15b** below, for example). As the target firing rate increases, a greater change in firing rate is predicted for the same change in fluorescence (**Supp fig 15c**). For example, a 0.50 ∆F/F change would generate a 1.9 Hz ramp with a 4 Hz target firing rate, a 2.8 Hz ramp with a 6 Hz target firing rate, and a 3.5 Hz ramp with an 8 Hz target firing rate (taken from linear model in **Supp fig 15c**). Nonetheless, the proportions of significantly position-modulated neurons that have increases or decreases in their inferred firing rates are similar across tuning rates (**Supp fig 15d,e**; 169/303 neurons (55.8%) are position-modulated at 4 Hz target firing rate; 184/303 (60.7%) are position-modulated at 6 Hz target firing rate; 196/303 (64.7%) are position-modulated at 8 Hz target firing rate; position modulation determined by significance in linear model with alpha p < 0.01).

**Supplementary figure 15. Upward and downward ramps in inferred spikes during reward approach in a virtual reality environment are detectable over a range of tuning parameters**. Results of spike inference during the virtual reality T-maze task in Fig when tuning parameters approximating mean firing rates of 4, 6, or 8 Hz are used. **a.** Mean inferred firing rate by position for example upward-ramping cells over a range of tuning parameters. **b.** Mean inferred firing rate by position for example downward-ramping cells over a range of tuning parameters. **c.** For each tuning parameter, scatterplots showing how change in observed GCaMP from beginning to end of maze for each neuron relates to change in inferred firing rate for that neuron. Each data point represents a single neuron and its mean change in observed GCaMP and inferred firing rate. Red line is linear least-squares fit; dotted region is 95% confidence of the fit. **d.** For each tuning parameter, histograms of changes of inferred firing rate for each neuron, color coded by whether a neuron’s probability of firing significantly decreased (red), increased (blue), or did not change (grey) with increases in maze position. **e.** For each tuning parameter, histograms of changes of the inferred firing rate slope for each neuron, color coded by whether a neuron’s probability of firing significantly decreased (red), increased (blue), or did not change (grey) with increases in maze position. Data includes cells expressing GCaMP6m and GCaMP6f.

We also show results of estimations on the VR maze data across the same range of decay parameters as used on the Pavlovian data (**Supp fig 16**, copied below). Varying the decay rate appears to have a relatively limited effect on both the magnitudes of inferred spike ramps and the proportions of significantly position-modulated neurons.

These figures detailing how results from the VR maze task change as a function of model parameters can also be found as **Supp. figs. 15-16** of the revised manuscript.

**Supplementary figure 16. Upward and downward ramps in inferred spikes during reward approach in a virtual reality environment are detectable over a range of decay rates**. Results of spike inference during the virtual reality T-maze task in Fig 4 when<http://www.sciweavers.org/tex2img.php?bc=Transparent&fc=Black&im=jpg&fs=100&ff=modern&edit=0&eq=%5Clambda#0> is selected to target a 6 Hz average estimated firing rate and the decay rate<http://www.sciweavers.org/tex2img.php?bc=Transparent&fc=Black&im=jpg&fs=100&ff=modern&edit=0&eq=%5Cgamma#0> is selected as the 25th percentile (low gamma), median, or 75th percentile (high gamma) estimated decay rate from the *in vitro* experiment (**Fig 1d**). **a.** Mean inferred firing rate by position for example upward-ramping cells over a range of decay rates. **b.** Mean inferred firing rate by position for example downward-ramping cells over a range of decay rates. **c.** For each decay rate, scatterplots showing how change in observed GCaMP from beginning to end of maze for each neuron relates to change in inferred firing rate for that neuron. Each data point represents a single neuron and its mean change in observed GCaMP and inferred firing rate. Red line is linear least-squares fit; dotted region is 95% confidence of the fit. **d.** For each decay rae, histograms of changes of inferred firing rate for each neuron, color coded by whether a neuron’s probability of firing significantly decreased (red), increased (blue), or did not change (grey) with increases in maze position. **e.** For each decay rate, histograms of changes of the inferred firing rate slope for each neuron, color coded by whether a neuron’s probability of firing significantly decreased (red), increased (blue), or did not change (grey) with increases in maze position. Data includes cells expressing GCaMP6m and GCaMP6f.

**In Figure 4, the authors show that positive and negative ramping spike rates can be recovered from ramping calcium signals in single neurons using their model. However, nothing is shown to validate their inference against electrophysiological data as in Figures 2 and 3. Does their spike inference model reveal features of the ramping that cannot be gleaned from the calcium signal alone (as for reward omission trials)? Other studies (i.e. Wei et al 2020) have argued that negative and positive ramps in single neuron spiking are differently represented in calcium signaling – does this model reconcile those discrepancies? Since the model parameters can vary across single neurons, can the authors show features of spike ramping at the population level that can be accurately recovered from their model (e.g. rates or proportions of positive and negative ramping neurons?)**

Thank you for these questions and the suggestion to better connect our spike inference results in Figure 4 to existing electrophysiology data. While we do not have a comparable electrophysiology dataset to directly confirm the predictions made by our spike inference, we can get a qualitative sense that our predictions are reasonable by making comparisons to previous papers in other tasks.

For example, Wei et al. (2020) note that “small fractional changes in spike rate can appear as prominent ramping in ∆F/F…” This observation is consistent with our findings: even in cells with large magnitude calcium ramps, we estimate relatively modest changes in firing rate. For example, on average a 0.50 ∆F/F change over the course of the maze corresponds to just a 2.8 Hz increase in firing rate (see **Fig. 4l**).

In addition, Kim et al. (2021), who performed electrophysiology recordings from dopamine neurons during a reward approach task in virtual reality, note that on average “spiking activity exhibited a positive ramp (slope, 0.16 ± 0.04 spikes/s^2^).” They also observe diversity in ramping, with both upward and downward ramping neurons. Most of the positive ramps they measure have slopes of <1.0 spikes/s^2^, and all of the negative ramps have slopes >-1.0 spikes/s^2^. We are able to calculate similar ramp slopes in our data. Indeed, the vast majority of ramps we measure are within this range (as seen in new panel **Fig. 4m**), with most positive ramps <0.5 spikes/s^2^, and most negative ramps >-0.5 spikes/s^2^. The group mean slope in our data set (0.04 spikes/s^2^) was smaller than that observed in the Kim et al. experiment, but this difference could very well be explained by differences in behavioral paradigms. Nonetheless, in the absence of a truly matching electrophysiology dataset, we can still conclude that our estimated slopes seem to be of similar magnitudes to those observed via electrophysiology is a promising sign that our spike inference method is able to recover appropriately sized ramps in spikes.

Regarding gleaning information from spike inference that was not obvious from the calcium signal alone, without spike inference it was unclear whether ramps in Ca++ would always correspond to ramps in inferred spikes. For example, a negative ramp could have been the product of slow decay of the calcium signal following a phasic activity at the beginning of the trial. Therefore, the prediction that ramping calcium corresponds closely to ramping spikes was an interesting observation, and also qualitatively consistent with the Kim et al. results on DA neuron ramping properties, as we mention above.

We appreciate the point that model parameters can vary across neurons. Our inference uses one set of parameters for all neurons, and it is possible that the recovery of ramping neurons might be sensitive to model parameters. To better understand how model parameters affect recovered ramps in inferred spikes, we performed inference using a range of tuning parameters that correspond to physiologically relevant mean firing rates (4, 6, and 8 Hz; **Supp. fig. 15**) and decay rates that correspond to the interquartile range observed in the in vitro experiments (**Supp. fig. 16**). In general, we find that our inference recovers similar proportions of ramping neurons when using these alternate model parameters. We do note that the magnitude of these ramps appears to increase as the tuning parameter’s target firing rate increases (**Supp. fig. 15d**), and thus slightly more neurons with significant position modulation are detected.

**Other points**

**a) In Fig. 1f, a tau value of 1 sec is chosen for all the curves. How much improvement is there if the actual fitted tau for each cell is used?**

To clarify, the tau value reflects a parameter used in the calculation of the van Rossum distance. We believe the reviewer may have meant to refer to decay rate<http://www.sciweavers.org/tex2img.php?bc=Transparent&fc=Black&im=jpg&fs=100&ff=modern&edit=0&eq=%5Cgamma#0> (which can also be interpreted in units of time, as we do in **Fig. 1e**). To further clarify, rather than use a decay rate that corresponds to a 1 second half life for all recordings, we use the median decay rate<http://www.sciweavers.org/tex2img.php?bc=Transparent&fc=Black&im=jpg&fs=100&ff=modern&edit=0&eq=%5Cgamma#0> for each experimental condition (as shown in **Fig. 1d**) for the van Rossum estimates in **Fig. 1f**.

The reviewer’s point that the quality of the spike estimation may vary based on the decay rate used is important. We believe our new panel **Fig. 1g**, in which we show how the van Rossum distance varies for each recording across a large range of possible decay rates, addresses this concern. In general, we see that the van Rossum distances tend to be much less dependent on the choice of decay rate<https://www.codecogs.com/eqnedit.php?latex=%5Cgamma#0> than on the choice of target firing rate<https://www.codecogs.com/eqnedit.php?latex=%5Clambda#0>.

**b) Also as a supplement for Fig. 1f, it would be helpful to the reader to illustrate what a difference of 5000 in the van Rossum distance means for the raster plot**

Thanks for this suggestion. We agree this is helpful information, especially given that by selecting a tuning parameter to approximate a mean 6 Hz firing rate in the in vivo experiments, we are likely misestimating the overall firing rate of some neurons.

To this end, we have added **Supp. fig. 6** to the revised manuscript. This figure contains examples of both a 6f and 6m recording at 37C. Each example features three types of estimates: one where we intentionally underestimate the observed firing rate, one where we match the tuning parameter to the observed firing rate, and one where we overestimate the firing rate. These misestimations generate large van Rossum distances (around 2000-9000), but appear to still capture tonic and phasic activity with some success.

**Supplementary figure 6. Example traces of inference results with large van Rossum distances resulting from misestimation of observed firing rate. a-c**. Examples of spike inference when tuning parameters are selected to underestimate (**a**), match (**b**), or overestimate (**c**) the observed firing rate of a neuron expressing GCaMP6f. **d-f**. Examples of spike inference when tuning parameters are selected to underestimate (**d**), match (**e**), or overestimate () the observed firing rate of a neuron expressing GCaMP6m. In both GCaMP6f and GCaMP6m examples, the van Rossum distance is lowest when the tuning parameter is selected to match the firing rate, but under- and overestimates appear to capture both tonic and phasic activity patterns.

**c) In the Methods, it is not clearly stated how long after the virus injection surgery the authors waited before performing imaging studies.**

Thank you for pointing this out. We have updated the Methods subsection “Animals and Surgery” to note that we waited 3-4 weeks after virus injection surgery before performing imaging studies.

**d) In the Methods, the authors comment on the need to precisely align the animal on the sphere. The “custom alignment tool” referred to is not particularly transparent. For reproducibility by others, clarifying what this tool is and how it is used would be useful.**

We have removed irrelevant details on the previously published data collection, since this was a reanalysis of that data and a detailed description of the data and methods are found in the original paper.

**e) To account for the rise time kinetics of the indicator, the authors state that they shift the inferred spike times by 4 time steps: some discussion of whether this shift is consistent among the indicators or neurons might be useful, along with a discussion on how this shift might be tuned, and why it is optimal to consider this potential third free parameter in this way rather than build in a double exponential with a rise parameter, as is often used to model these calcium transients.**

Thanks for this question. We shift the inferred spike times by 4 time steps to account for the fact that our model is a bit misspecified. Under our model, a spike leads to an instantaneous increase in calcium and fluorescence; however, in practice, there is a bit of a delay, and the fluorescence rises slowly over the course of a few time steps following a spike.

In principle, we could consider the number of time steps by which we shift the inferred spike times as a tuning parameter. However, we do not see much benefit to doing so: in practice, a shift of 4 time steps works well. Furthermore, while it is in principle possible that the optimal number of time steps could vary across neurons, recordings, or experiments, we have not observed this in practice.

As an alternative to shifting the inferred spike times by 4 time steps, we could consider a more complex model in which the calcium follows a second-order (rather than a first-order) auto-regressive model. This would involve replacing the model<https://www.codecogs.com/eqnedit.php?latex=c_t%20%3D%20%5Cgamma%20c_%7Bt-1%7D%20%2B%20z_t#0> with the model<https://www.codecogs.com/eqnedit.php?latex=c_t%20%3D%20%5Cgamma_1%20c_%7Bt-1%7D%20%2B%20%5Cgamma_2%20c_%7Bt-2%7D%20%2B%20z_t#0>. With an appropriate choice of the parameters<https://www.codecogs.com/eqnedit.php?latex=%5Cgamma_1#0> and<https://www.codecogs.com/eqnedit.php?latex=%5Cgamma_2#0>, this model can allow for a slow rise in calcium and fluorescence following a spike. However, there are some major drawbacks to this approach:

- The fast algorithm for solving the optimization problem from Jewell et al. [(3)](https://paperpile.com/c/BQqrn1/O4Z7) cannot be applied to the second-order auto-regressive model. Thus, we would need to apply a greedy algorithm that would not find the global optimum, as in Friedrich et al. [(4)](https://paperpile.com/c/BQqrn1/tIoB).
- It would be hard to tune the two parameters<https://www.codecogs.com/eqnedit.php?latex=%5Cgamma_1#0> and<https://www.codecogs.com/eqnedit.php?latex=%5Cgamma_2#0>. In particular, the simple approach used in this paper to tune<https://www.codecogs.com/eqnedit.php?latex=%5Cgamma#0> by fitting an exponential decay model to a region with no spikes could not be applied.
- While<https://www.codecogs.com/eqnedit.php?latex=%5Cgamma#0> in the current model has a simple interpretation as the decay rate associated with the calcium indicator,<https://www.codecogs.com/eqnedit.php?latex=%5Cgamma_1#0> and<https://www.codecogs.com/eqnedit.php?latex=%5Cgamma_2#0> would be much less interpretable.
- The bias-variance trade-off tells us that fitting a more complex model will reduce bias, but may potentially lead to a substantial increase in variance. We suspect that in this case, we will not see much practical improvement due to fitting this more complex model.

For these reasons, we do not pursue the second-order auto-regressive model approach in this paper.

In general, we believe the 4-step time shift is a sensible selection for both GCaMP sensors. This shift corresponds to roughly a 0.06 s time shift, which appears to be in the range with the reported rise times in cortical cells expressing GCaMP6m and GCaMP6f in Chen et al. [(5)](https://paperpile.com/c/BQqrn1/DkLQ).

We see in our own data that a 4-step time shift generates similar results as do 5- or 6-step shifts in spike inference performance as measured by van Rossum distance. There is no difference between the 4-step and 5-step shifts in the GCaMP6f 37C condition (p = 0.137 for 5-step shift; p = 0.475 for 6-step shift; paired t-test), though VR distances are lower for 5- and 6-step shifts for the GCaMP6m 37C condition (p = 0.006 for 5-step shift; p = 0.016 for 6-step shift; paired t-test).

**van Rossum distance as a function of number of points shifted with step adjustment**. For each recording in the 6f 37C and 6m 37C data, the van Rossum distance as a function of the number of points shifted when the group median decay rate is used and the tuning parameter is selected to match the observed firing rate. A 4-point shift was used in our spike inference, but van Rossum distances are comparable when 5- or 6-point shifts are used. This figure is not included in the manuscript.

We also examined how GCaMP kinetics compared to observed spike times, since the time shift is ultimately intended to best reflect the difference between a spike time and the corresponding increase in observed calcium fluorescence. On average, we see similar rise kinetics between the GCaMP6f and GCaMP6m experiments at 37C. The slope of the increase in calcium fluorescence appears greatest at a time point corresponding to a 4-step shift, although it is clear from these plots that a 5-step shift would also be a reasonable selection.

**Mean observed fluorescence in relation to isolated spikes in 37C conditions**. The mean fluorescence across all bursty recordings for the 6f (green) and 6m (orange) conditions, with respect to observed spike times. Vertical grey lines represent the time between the real spike and where an inferred spike would be detected prior to the step adjustment. Only spikes where there was at least a 200 ms period of no spikes prior were used for analysis. This figure is not included in the manuscript.

**f) The type of GCaMP (m or f) used for imaging experiments should be indicated in each figure legend if possible**

We have updated our figure legends accordingly. In brief, data from the Pavlovian experiments (**Figs. 2-3**) was all from cells expressing GCaMP6f, whereas data from the VR maze experiment (**Fig. 4**) was from a mix of cells expressing either GCaMP6f or GCaMP6m.

**g) More discussion of the applications and limitations of this model to future in-vivo dopamine neuron recordings at the single neuron and population levels would be beneficial for potential adopters.**

Good point. We clarify that this approach should be particularly valuable when there are events that are close to each other in time that modulate the neural signal, to properly account for the slow decay of the Ca++ signal and identify correlates of each event. In addition, we have added a paragraph to the **Limitations of spike inference** in VTA DA neurons subsection of the **Discussion** mentioning that, since the overall firing rate is determined by the user’s selection of target firing rate for the tuning parameter, users should consider conducting inference over a range of possible target firing rates, and results should ideally be interpreted with respect to the overall firing rate (by baseline subtracting phasic responses, for example).

**h) Do model parameters differ across different midbrain regions? The in-vitro data may be too limited to address this point, but some discussion may be pertinent.**

Thank you for this question. It is likely that tuning parameter<https://www.codecogs.com/eqnedit.php?latex=%5Clambda#0> varies across midbrain regions given that different cell types have different firing rates. In the revised Discussion, we now advise future adopters to consider using a range of tuning parameters and interpret changes in firing rate with respect to the overall firing rate to account for the possibility of misestimating the true firing rate of the measured population. On the other hand, our results (see **Fig. 1g** of the revised manuscript) imply that the exact choice of decay rate parameters is not essential.

**Journal Requirements:**

**When submitting your revision, we need you to address these additional requirements.**

**1. Please ensure that your manuscript meets PLOS ONE's style requirements, including those for file naming. The PLOS ONE style templates can be found at**

<https://journals.plos.org/plosone/s/file?id=wjVg/PLOSOne_formatting_sample_main_body.pdf> and

<https://journals.plos.org/plosone/s/file?id=ba62/PLOSOne_formatting_sample_title_authors_affiliations.pdf>

**2. We note that you have included the phrase “data not shown” in your manuscript. Unfortunately, this does not meet our data sharing requirements. PLOS does not permit references to inaccessible data. We require that authors provide all relevant data within the paper, Supporting Information files, or in an acceptable, public repository. Please add a citation to support this phrase or upload the data that corresponds with these findings to a stable repository (such as Figshare or Dryad) and provide and URLs, DOIs, or accession numbers that may be used to access these data. Or, if the data are not a core part of the research being presented in your study, we ask that you remove the phrase that refers to these data.**

We added a new supplementary figure (**Supp. fig. 11** in the revised manuscript) to show the data referred to in this one instance where we had used “data not shown.” We deleted this use of “data not shown,” and now point to that supplementary figure in the revised manuscript.

**3. We note that you have stated that you will provide repository information for your data at acceptance. Should your manuscript be accepted for publication, we will hold it until you provide the relevant accession numbers or DOIs necessary to access your data. If you wish to make changes to your Data Availability statement, please describe these changes in your cover letter and we will update your Data Availability statement to reflect the information you provide.**

We will make the data available.

**REFERENCES**

1.	[Khaliq ZM, Bean BP. Pacemaking in dopaminergic ventral tegmental area neurons: depolarizing drive from background and voltage-dependent sodium conductances. J Neurosci. 2010 May 26;30(21):7401–13.](http://paperpile.com/b/BQqrn1/D39s)

2.	[Mereu G, Lilliu V, Casula A, Vargiu PF, Diana M, Musa A, et al. Spontaneous bursting activity of dopaminergic neurons in midbrain slices from immature rats: role of N-methyl-D-aspartate receptors. Neuroscience. 1997 Apr;77(4):1029–36.](http://paperpile.com/b/BQqrn1/DTgX)

3.	[Jewell SW, Hocking TD, Fearnhead P, Witten DM. Fast nonconvex deconvolution of calcium imaging data. Biostatistics [Internet]. 2019 Feb 8; Available from:](http://paperpile.com/b/BQqrn1/O4Z7) <http://dx.doi.org/10.1093/biostatistics/kxy083>

4.	[Friedrich J, Zhou P, Paninski L. Fast online deconvolution of calcium imaging data. PLoS Comput Biol. 2017 Mar;13(3):e1005423.](http://paperpile.com/b/BQqrn1/tIoB)

5.	[Chen T-W, Wardill TJ, Sun Y, Pulver SR, Renninger SL, Baohan A, et al. Ultrasensitive fluorescent proteins for imaging neuronal activity. Nature. 2013 Jul 18;499(7458):295–300.](http://paperpile.com/b/BQqrn1/DkLQ)
